# Sex Differences in the Spatial Behavior Functions of Adult-Born Neurons in Rats

**DOI:** 10.1523/ENEURO.0054-22.2022

**Published:** 2022-05-11

**Authors:** Timothy P. O’Leary, Baran Askari, Bonnie H. Lee, Kathryn Darby, Cypress Knudson, Alyssa M. Ash, Desiree R. Seib, Delane F. Espinueva, Jason S. Snyder

**Affiliations:** Department of Psychology, Djavad Mowafaghian Centre for Brain Health, University of British Columbia, Vancouver, British Columbia, V6T 2B5, Canada

**Keywords:** hippocampus, learning and memory, neurogenesis, plasticity, sex differences, strategy

## Abstract

Adult neurogenesis modifies hippocampal circuits and behavior, but removing newborn neurons does not consistently alter spatial processing, a core function of the hippocampus. Additionally, little is known about sex differences in neurogenesis since few studies have compared males and females. Since adult-born neurons regulate the stress response, we hypothesized that spatial functions may be more prominent under aversive conditions and may differ between males and females given sex differences in stress responding. We therefore trained intact and neurogenesis-deficient rats in the spatial water maze at temperatures that vary in their degree of aversiveness. In the standard water maze, ablating neurogenesis did not alter spatial learning in either sex. However, in cold water, ablating neurogenesis had divergent sex-dependent effects: relative to intact rats, male neurogenesis-deficient rats were slower to escape the maze and female neurogenesis-deficient rats were faster. Neurogenesis promoted temperature-related changes in search strategy in females, but it promoted search strategy stability in males. Females displayed greater recruitment (Fos expression) of the dorsal hippocampus than males, particularly in cold water. However, blocking neurogenesis did not alter Fos expression in either sex. Finally, morphologic analyses revealed greater experience-dependent plasticity in males. Adult-born neurons in males and females had similar morphology at baseline but training increased spine density and reduced presynaptic terminal size, specifically in males. Collectively, these findings indicate that adult-born neurons contribute to spatial learning in stressful conditions and they provide new evidence for sex differences in their behavioral functions.

## Significance Statement

New neurons are added in adulthood to the hippocampus, a structure involved in memory. However, the behavioral functions of adult-born neurons remain unclear. Since new neurons also regulate stress-related behavior, we tested whether they may be important for learning under stress in rats, and we included both males and females since there are known sex differences in the stress response. While blocking neurogenesis caused males to learn slower, it caused females to learn faster, in an aversive water maze task. Learning in aversive conditions also altered the structure of newborn neurons in males but not females. These results indicate that newborn neurons may play distinct roles in cognition and mental health in males and females.

## Introduction

Adult hippocampal neurogenesis has been implicated in many of the mnemonic functions of the hippocampus, including memory for temporal events (Shors et al., 2001b, 2002; Seo et al., 2015), locations (Clelland et al., 2009), contexts (Saxe et al., 2006; Winocur et al., 2006), objects (Jessberger et al., 2009; Denny et al., 2012), probabilistic rewards (Seib et al., 2020), conspecifics (Cope et al., 2020), as well as the consolidation (Kitamura et al., 2009; Kumar et al., 2020) and forgetting (Akers et al., 2014) of memory. While spatial memory functions may be particularly apparent in conditions that maximize conflict or interference, such as when a goal changes location (Garthe et al., 2009; Burghardt et al., 2012; Swan et al., 2014; Yu et al., 2019), it is notable that many studies have failed to find a role for new neurons in learning and short-term reference memory in the spatial water maze, a task that is highly sensitive to hippocampal disruption (Shors et al., 2002; Madsen et al., 2003; Raber et al., 2004; Snyder et al., 2005; Saxe et al., 2006; Jessberger et al., 2009; Blaiss et al., 2011; Groves et al., 2013; Nickell et al., 2020).

A relatively independent body of work has focused on the role of neurogenesis in emotional and stress-related behavior, finding that neurogenesis buffers the endocrine response to acute stressors and reduces depressive-like and anxiety-like behavior (Revest et al., 2009; Lagace et al., 2010; Snyder et al., 2011; Surget et al., 2011; Lehmann et al., 2013; Anacker et al., 2018; Schoenfeld et al., 2019). Since stress and emotion potently modulate learning and memory (Bangasser and Shors, 2010; Roozendaal and McGaugh, 2011), here we hypothesized that a role for neurogenesis in spatial learning may become particularly apparent in more aversive conditions. Consistent with this possibility, a small number of studies have found that neurogenesis does alter behavior in memory tasks depending on the aversiveness of conditioned and unconditioned stimuli that are present (Drew et al., 2010; Seo et al., 2015; Schoenfeld et al., 2021).

Stress-related disorders such as anxiety, PTSD, and depression impact a substantial fraction of the population and these disorders affect women to a greater extent than men. Together with the data from rodents, this suggests that neurogenesis functions in stress may vary depending on sex and gender (Kessler et al., 2012). Indeed, there are known sex differences in the rates of addition (Chow et al., 2013), maturation (Yagi et al., 2020) and activation of adult-born neurons (Yagi et al., 2016). Furthermore, there are sex differences in hippocampal plasticity (Juraska et al., 1985; Warren et al., 1995; Shors et al., 2001a; Scharfman and MacLusky, 2014; Le et al., 2022) and behavioral responses to acute and chronic stress (Luine, 2002; Conrad et al., 2004; Bangasser and Shors, 2007). However, as is the case in neuroscience more broadly (Beery and Zucker, 2011), the majority of neurogenesis studies have focused on males (Huckleberry and Shansky, 2021). Many do not report/analyze data by sex and >20% of studies do not report the sex of their subjects (Knudson et al., 2022). To our knowledge, only two studies have reported behavioral sex differences in neurogenesis-deficient animals. One study reported sex differences in neurogenic modulation of the hypothalamic-pituitary-adrenal (HPA) response (Silveira‐Rosa et al., 2021), although this rat model is confounded by neurogenesis-independent effects on emotion (Groves et al., 2013). A second study found that adult neurogenesis buffers the effects of early life stress on subsequent anxiety-like behavior, selectively in male mice (Waters et al., 2022). Whether adult neurogenesis differentially regulates behavior across the sexes in learning situations, however, remains unknown.

To address these outstanding issues we used a pharmacogenetic GFAP-TK (TK) rat model to block adult neurogenesis (Snyder et al., 2016), and tested male and female rats in the water maze at warm (25°C, standard) or cold (16°C, more aversive/stressful) temperatures. Consistent with previous work, neurogenesis-deficient rats were unimpaired at standard water maze temperatures. However, cold water testing revealed striking sex differences in the behavioral function of adult-born neurons, and also elicited distinct dorsoventral patterns of hippocampal recruitment and new neuron plasticity in males and females.

## Materials and Methods

### Subjects

This study used male and female transgenic GFAP-TK (TK) and wild-type (WT) littermate rats on a Long–Evans background (Snyder et al., 2016). Here, a GFAP promoter drives expression of herpes simplex virus thymidine kinase in radial-glial precursor cells, enabling these cells to be killed when rats are treated with valganciclovir and the cells attempt mitosis. Rats were bred in-house, by crossing heterozygous transgenic females with WT males. After weaning (postnatal day 21) rats were housed in same-sex groups of two to three in polyurethane cages (48 × 27 × 20 cm), with aspen chip bedding, a polycarbonate tube for enrichment, and ad-libitum access to food and water. Animals were housed under a 12/12 h light/dark cycle, and all testing was completed during the light phase. Rats were genotyped via PCR after weaning and, therefore, housed randomly with respect to genotype. Before all experiments, animals were handled 5 min/d for 5 d. Experimental procedures were approved by the University of British Columbia Animal Care Committee and followed guidelines from the Canadian Council of Animal Care on the ethical treatment of animals.

### Valganciclovir treatment and untreated controls

**Figure 1.**
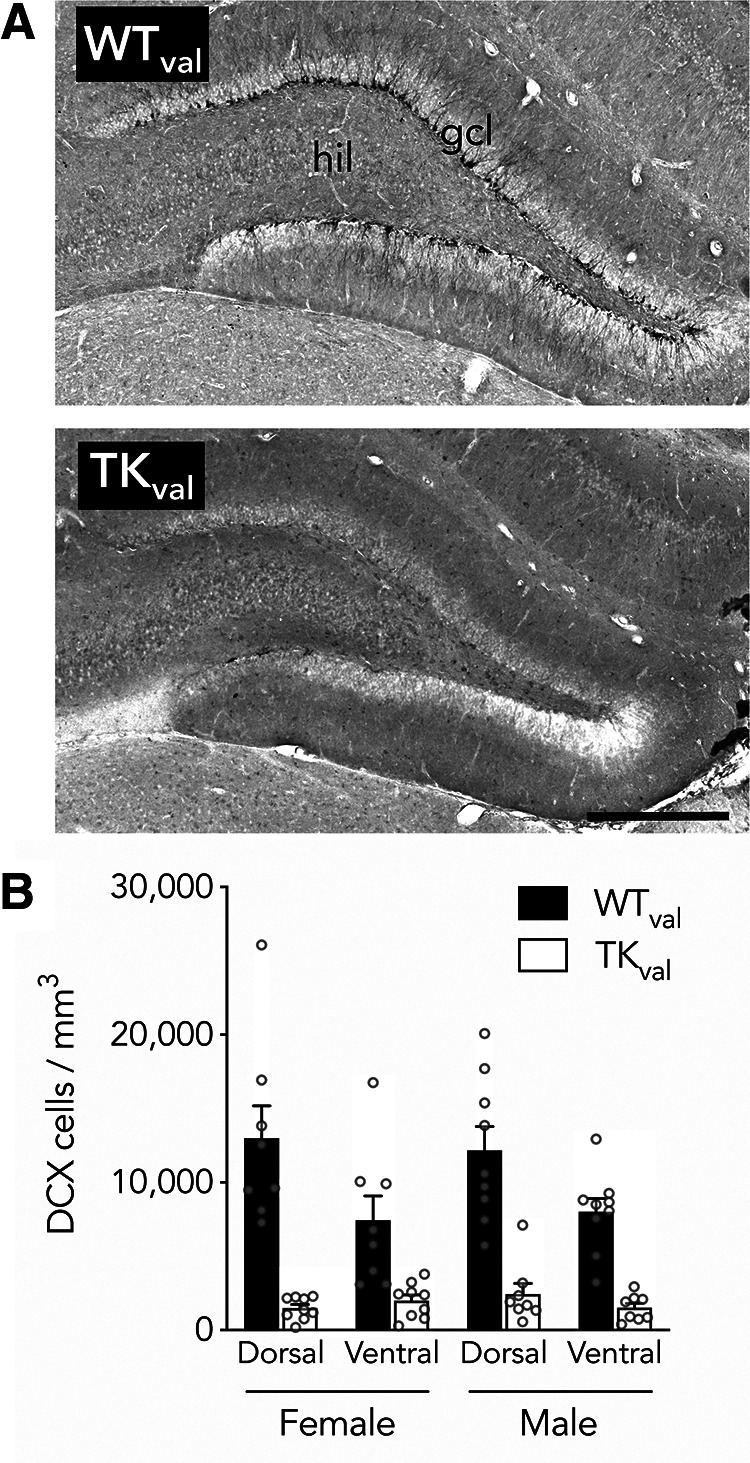
Reduced neurogenesis in valganciclovir-treated GFAP-TK rats. ***A***, Representative immunostaining for the immature neuronal marker, DCX, in WT (top) and TK (bottom) rats (here, both female). hil, hilus; gcl, granule cell layer. Scale bar: 500 μm. ***B***, Neurogenesis was suppressed along the dorsoventral axis of both male and female rats (effect of genotype: *F*_(1,30)_ = 58, *p* < 0.0001, η_p_^2^ = 0.66; effect of sex: *F*_(1,30)_ = 0.0, *p* = 0.96, η_p_^2^ = 0; effect of dorsoventral subregion: *F*_(1,30)_ = 28, *p* < 0.0001, η_p_^2^ = 0.48; sex interactions all *p* > 0.15). Bars reflect mean ± SE. Detailed statistical analyses and full underlying dataset for this and all other figures can be found in Extended Data Figure 1-1.

**Figure 2.**
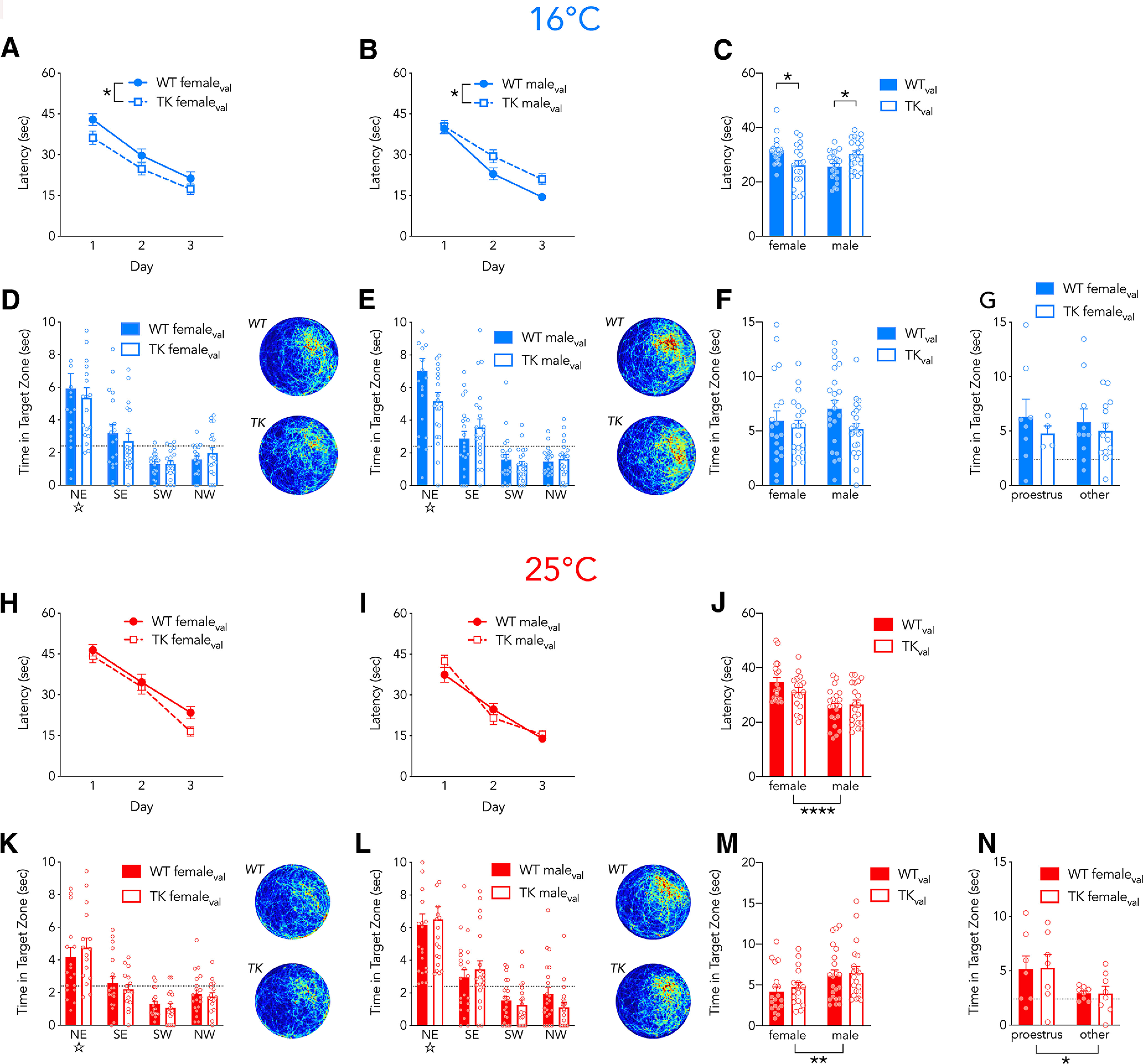
Newborn neurons modulate spatial learning in a sex-dependent and temperature-dependent fashion. ***A–G***, In the 16°C water maze, valganciclovir-treated female (***A***) and male (***B***) rats reached the platform faster with successive days of training (effect of day, *F*_(2,156)_ = 105, *p* < 0.0001, η_p_^2^ = 0.44). There was no main effect of sex (*F*_(1,78)_ = 0.3, *p* = 0.6, η_p_^2^ = 0.004) or genotype (*F*_(1,78)_ = 0.1, *p* = 0.8, η_p_^2^ = 0), but there was a significant sex × genotype interaction (*F*_(1,78)_ = 15, *p* = 0.0003, η_p_^2^ = 0.16). Male WT rats reached the platform faster than male TK rats (*p* = 0.01, g = 0.71), female WT rats reached the platform slower than female TK rats (*p* = 0.01, g_s_ = 0.80) and male WT rats reached the platform faster than female WT rats (*p* = 0.005, g_s_ = 1.09). ***C***, Summary of average acquisition latencies in females and males. On the probe trial, female (***D***) and male (***E***) rats preferentially searched in the target (NE) zone where the platform was located during training. Dotted line indicates chance performance. Male TK rats tended to search less in the target zone but effects of sex and genotype were not significant (effect of genotype, *F*_(1,78)_ = 2.8, *p* = 0.097, η_p_^2^ = 0.058; effect of sex, *F*_(1,78)_ = 0.4, *p* = 0.5, η_p_^2^ = 0.006; interaction, *F*_(1,78)_ = 0.8, *p* = 0.4, η_p_^2^ = 0.011). ***F***, Summary of time spent in the target zone of the probe trial for females and males. ***G***, Estrous stage did not influence performance on the probe trial (effect of genotype, *F*_(1,32)_ = 0, *p* = 0.9; effect of estrous stage, *F*_(1,32)_ = 0, *p* = 0.9; interaction, *F*_(1,32)_ = 0, *p* = 0.7). ***H–N***, In the 25°C water maze, valganciclovir-treated female (***H***) and male (***I***) rats reached the platform faster with successive days of training (effect of day, *F*_(2,150)_ = 162, *p* < 0.0001, η_p_^2^ = 0.68). Males reached the platform faster than females (effect of sex, *F*_(1,75)_ = 20, *p* < 0.0001, η_p_^2^ = 0.21), but there was no difference between WT and TK rats (effect of genotype, *F*_(1,75)_ = 0.6, *p* = 0.4, η_p_^2^ = 0.01) and no significant interactions between day, sex, and genotype (all *p* > 0.09). ***J***, Summary of average trial acquisition latency. On the probe trial, female (***K***) and male (***L***) rats preferentially searched in the target zone. WT and TK rats did not differ on the probe trial but males spent more time searching in the target zone (effect of genotype, *F*_(1,73)_ = 0.5, *p* = 0.5, η_p_^2^ = 0.007; effect of sex, *F*_(1,73)_ = 8, *p* = 0.0075, η_p_^2^ = 0.094; interaction, *F*_(1,73)_ = 0, *p* = 0.9, η_p_^2^ = 0.00). ***M***, Summary of probe trial target zone search time for males and females. ***N***, Females in proestrus displayed better memory on the probe trial than rats in other phases of the estrous cycle (effect of genotype, *F*_(1,26)_ = 0, *p* = 0.9; effect of estrous stage, *F*_(1,26)_ = 6.5, *p* = 0.02; interaction, *F*_(1,26)_ = 0, *p* = 0.8). *p < 0.05, **p < 0.01, ****p < 0.0001. *N* = 17–22 per group. Bars and symbols reflect mean ± SE. All heat maps are scaled equivalently.

**Figure 3.**
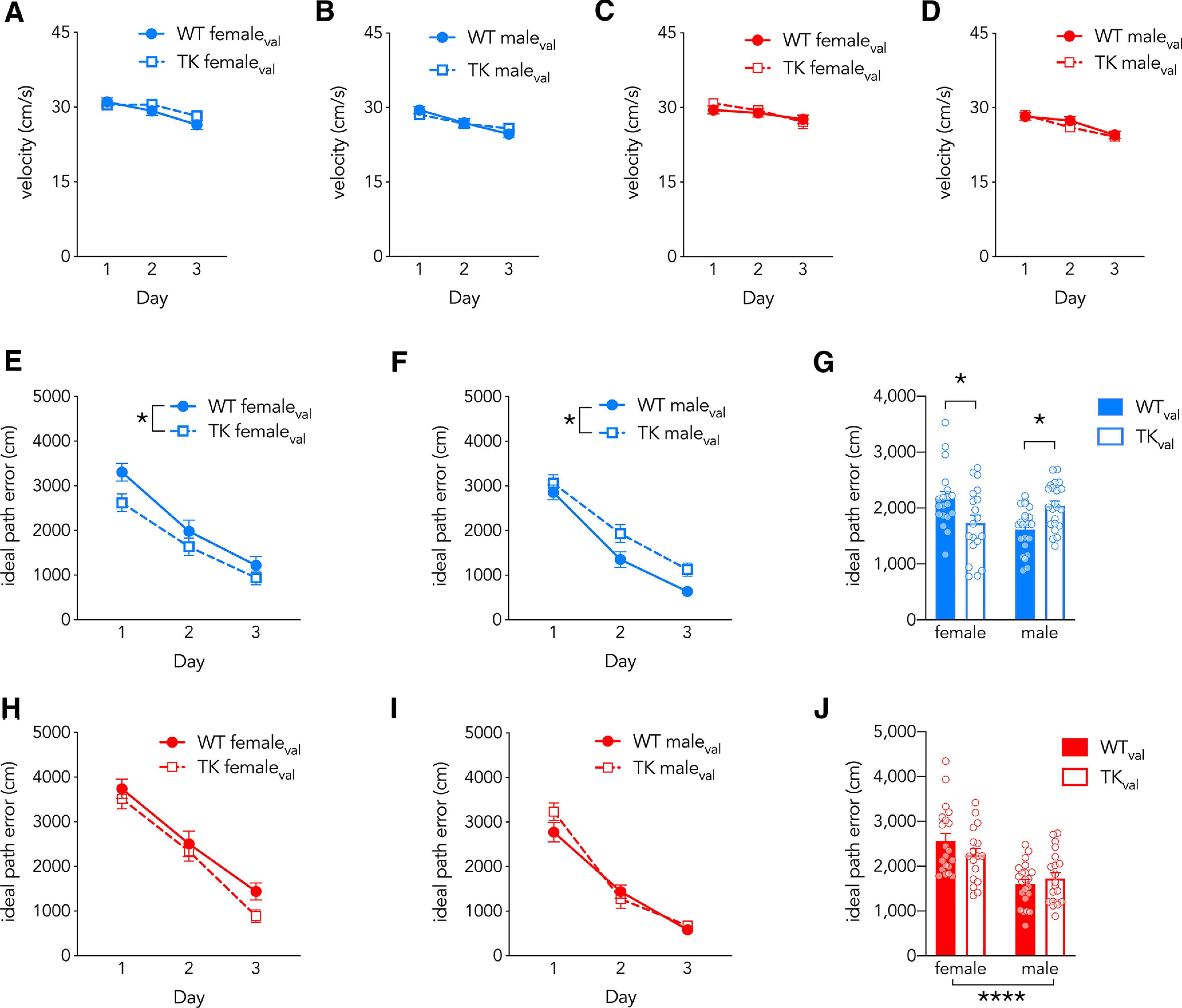
Additional measures of water maze performance in valganciclovir-treated rats. ***A***, ***B***, In the 16°C water maze, females swam faster than males, swim speed declined over days, and there were no differences between WT and TK rats (effect of sex: *F*_(1,78)_ = 8, *p* = 0.006, η_p_^2^ = 0.09; effect of day: *F*_(2,156)_ = 32, *p* < 0.0001, η_p_^2^ = 0.29; effect of genotype: *F*_(1,78)_ = 0.1, *p* = 0.7, η_p_^2^ = 0; all interactions *p* > 0.05). ***C***, ***D***, In the 25°C water maze, females swam faster than males, swim speed declined over days, and there were no differences between WT and TK rats (effect of sex: *F*_(1,75)_ = 13, η_p_^2^ = 0.15, *p* = 0.0006; effect of day: *F*_(2,150)_ = 34, *p* < 0.0001, η_p_^2^ = 0.31; effect of genotype: *F*_(1,75)_ = 0, *p* = 1, η_p_^2^ = 0; all interactions *p* > 0.23). ***E***, ***F***, In the 16°C water maze, ideal path error decreased over days (effect of genotype, *F*_(1,78)_ = 0.0, *p* = 0.99, η_p_^2^ = 0; effect of day, *F*_(2,156)_ = 127, *p* < 0.0001, η_p_^2^ = 0.62; effect of sex, *F*_(1,78)_ = 1.3, *p* = 0.3, η_p_^2^ = 0.02). Blocking neurogenesis increased ideal path error in males but decreased it in females (genotype × sex interaction: *F*_(1,78)_ = 16, *p* = 0.0002, η_p_^2^ = 0.16; male WT vs male TK: *p* = 0.01, g_s_ = 1.05; female WT vs female TK: *p* = 0.02, g_s_ = 0.72). ***G***, Average ideal path error scores during training. ***H***, ***I***, In the 25°C water maze, ideal path error was not different between genotypes, but decreased over days and was lower for males than females (effect of genotype, *F*_(1,75)_ = 0.4, *p* = 0.5, η_p_^2^ = 0; effect of day, *F*_(2,150)_ = 215, *p* < 0.0001, η_p_^2^ = 0.74; effect of sex, *F*_(1,75)_ = 29, *p* < 0.0001, η_p_^2^ = 0.28; all interactions, *p* ≥ 0.05). ***J***, Average trial ideal path error scores during training. *N* = 17–22 per group. Symbols reflect mean ± SE. *p < 0.05, ****p < 0.0001.

**Figure 4.**
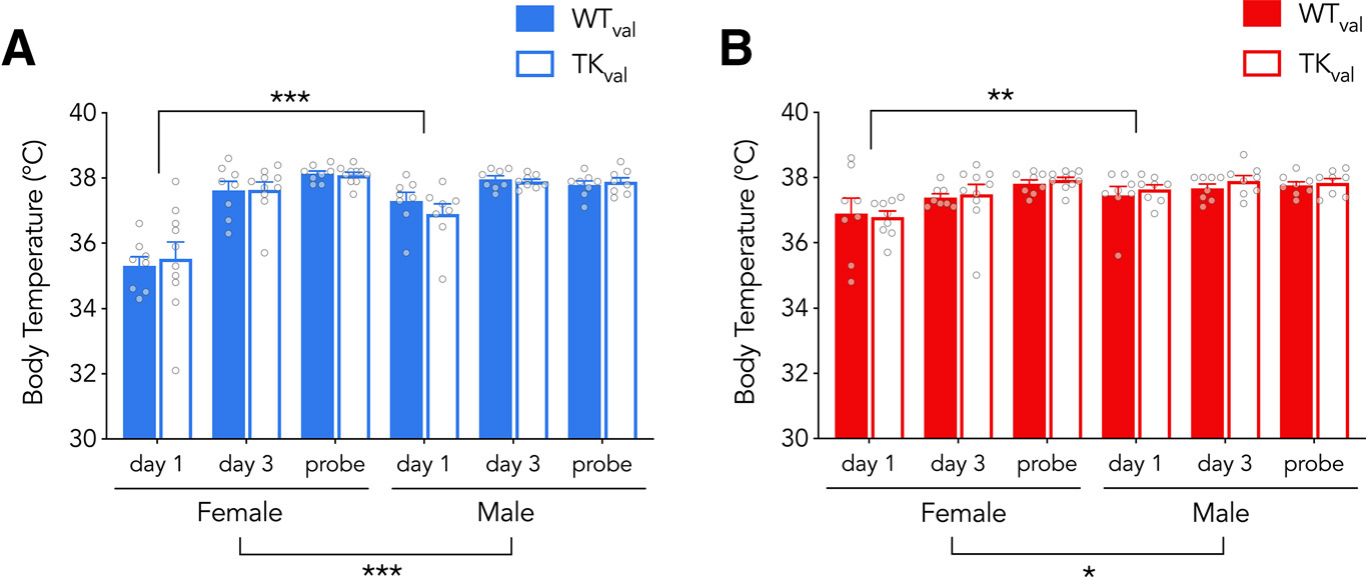
Body temperatures following testing. ***A***, At 16°C, in valganciclovir-treated rats, post-testing body temperatures were lowest on day 1, were lower on day 1 in females than in males but were not different between WT and TK rats (effect of day: *F*_(2,60)_ = 52, *p* < 0.0001, η_p_^2^ = 0.64; effect of sex: *F*_(1,30)_ = 14, *p* = 0.0008, η_p_^2^ = 0.32; effect of genotype: *F*_(1,30)_ = 0.04, *p* = 0.8, η_p_^2^ = 0; day × sex interaction: *F*_(2,60)_ = 15, *p* < 0.0001, η_p_^2^ = 0.33; all genotype interactions *p* > 0.5; day 1 vs day 3 and day 1 vs probe both *p* < 0.0001; day 1 female vs male: *p* < 0.001, g_s_ = 1.51; day 3 and probe female vs male: *p* > 0.5). ***B***, At 25°C, post-testing body temperatures were lowest on day 1, were lower on day 1 in females than in males but were not different between WT and TK rats (effect of day: *F*_(2,59)_ = 11, *p* < 0.0001, η_p_^2^ = 0.28; effect of sex: *F*_(1,30)_ = 4.8, *p* = 0.04, η_p_^2^ = 0.14; effect of genotype: *F*_(1,30)_ = 0.4, *p* = 0.5, η_p_^2^ = 0.01; all genotype interactions *p* > 0.6; day × sex interaction: *F*_(2,59)_ = 3.9, *p* = 0.03, η_p_^2^ = 0.12; day 1 vs day 3, *p* = 0.005, day 1 vs probe, *p* < 0.0001; day 1 female vs male: *p* = 0.005, g_s_ = 0.84, day 3 and probe female vs male both *p* > 0.27). Bars reflect mean ± SE. *p < 0.05, **p < 0.01, ***p < 0.001.

**Figure 5.**
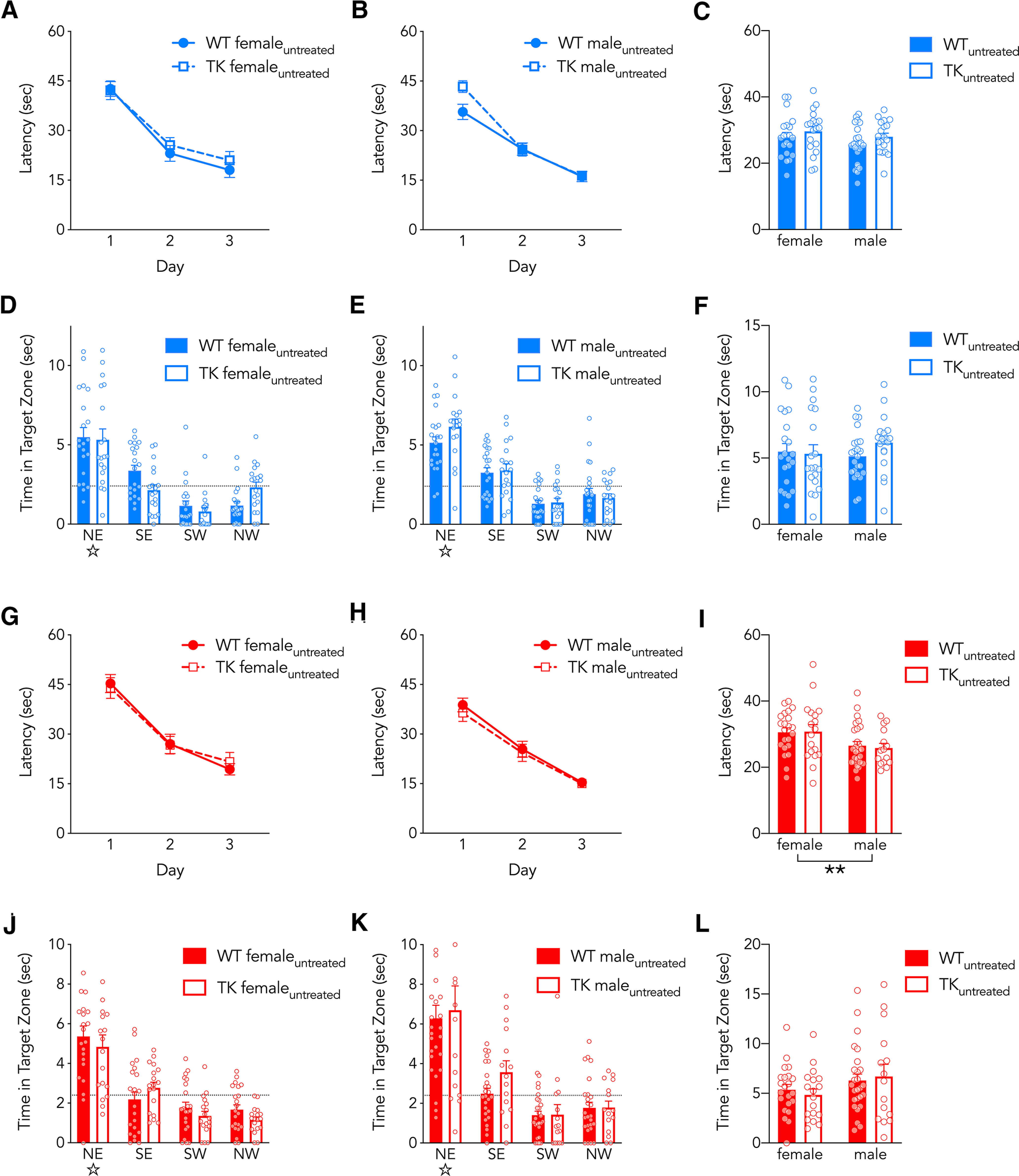
Water maze performance in WT and TK rats that were not treated with valganciclovir. ***A–C***, Spatial water maze learning at 16°C was similar in WT and TK rats (effect of day, *F*_(2,156)_ = 130, *p* < 0.0001, η_p_^2^ = 0.50; effect of sex, *F*_(1,78)_ = 2.6, *p* = 0.1, η_p_^2^ = 0.03; effect of genotype, *F*_(1,78)_ = 2.7, *p* = 0.1, η_p_^2^ = 0.03; interactions all *p* > 0.1). ***D–F***, 16°C probe trial performance was similar in WT and TK rats (effect of genotype, *F*_(1,78)_ = 0.6, *p* = 0.4 η_p_^2^ = 0; effect of sex, *F*_(1,78)_ = 0.2, *p* = 0.6, η_p_^2^ = 0; interaction, *F*_(1,78)_ = 1.2, *p* = 0.3, η_p_^2^ = 0.02). ***G–I***, Spatial water maze learning at 25°C was similar in WT and TK rats (effect of day, *F*_(2,154)_ = 103, *p* < 0.0001, η_p_^2^ = 0.57; effect of sex, *F*_(1,77)_ = 10, *p* = 0.0028, η_p_^2^ = 0.11; effect of genotype, *F*_(1,77)_ = 0.1, *p* = 0.7, η_p_^2^ = 0; interactions all *p* > 0.29). ***J–L***, 25°C probe trial performance was similar in WT and TK rats (effect of genotype, *F*_(1,77)_ = 0, *p* = 0.9, η_p_^2^ = 0; effect of sex, *F*_(1,77)_ = 3.4, *p* = 0.07, η_p_^2^ = 0.042; interaction, *F*_(1,77)_ = 0.3, *p* = 0.5 η_p_^2^ = 0). *N* = 15–26 per group. Bars and symbols indicate mean ± SE.

**Figure 6.**
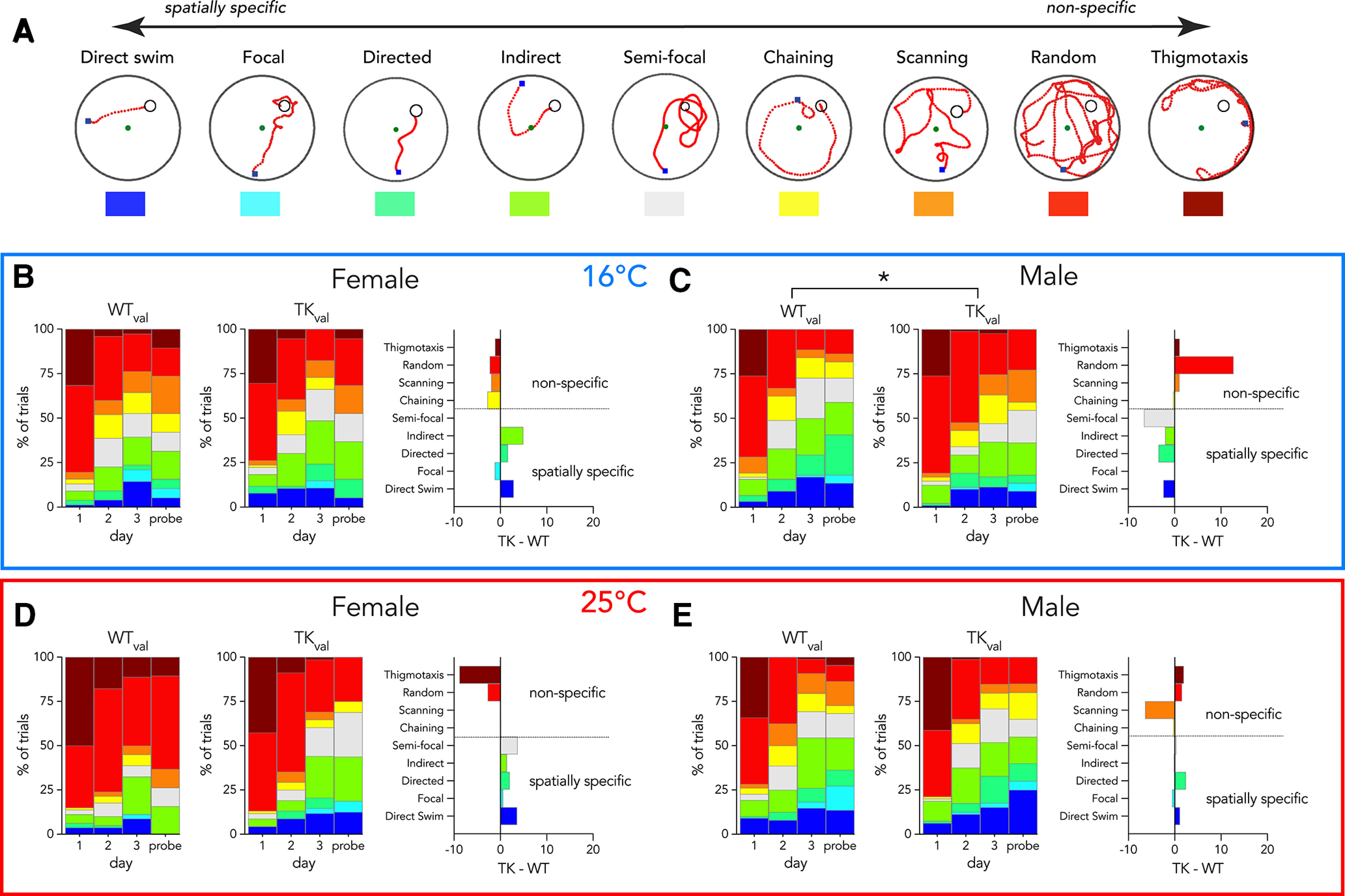
In the 16°C water maze, blocking neurogenesis reduces spatially-specific search in male rats. ***A***, Example trials illustrating various search strategies classified by Pathfinder, organized by degree of spatial specificity relative to the target. ***B***, ***C***, 16°C water maze strategies in valganciclovir-treated rats. ***B***, The distribution of strategies in female TK rats was not significantly different from female WT rats (χ^2^ = 7, *p* = 0.5, V = 0.44). Right-most graph shows difference scores for the various strategies. ***C***, Reducing neurogenesis significantly altered the distribution of strategies used by male rats at 16°C, demonstrated by the greater proportion of spatially nonspecific trials and the smaller proportion of spatially-specific trials (right; χ^2^ = 17, *p* = 0.02, V = 0.63). ***D***, ***E***, 25°C water maze strategies in valganciclovir-treated rats. ***D***, The distribution of strategies used by female TK rats was not significantly different from female WT rats (χ^2^ = 12, *p* = 0.15, V = 0.57). ***E***, Reducing neurogenesis did not alter the distribution of strategies used by male rats (χ^2^ = 11, *p* = 0.4, V = 0.52). *p < 0.05.

**Figure 7.**
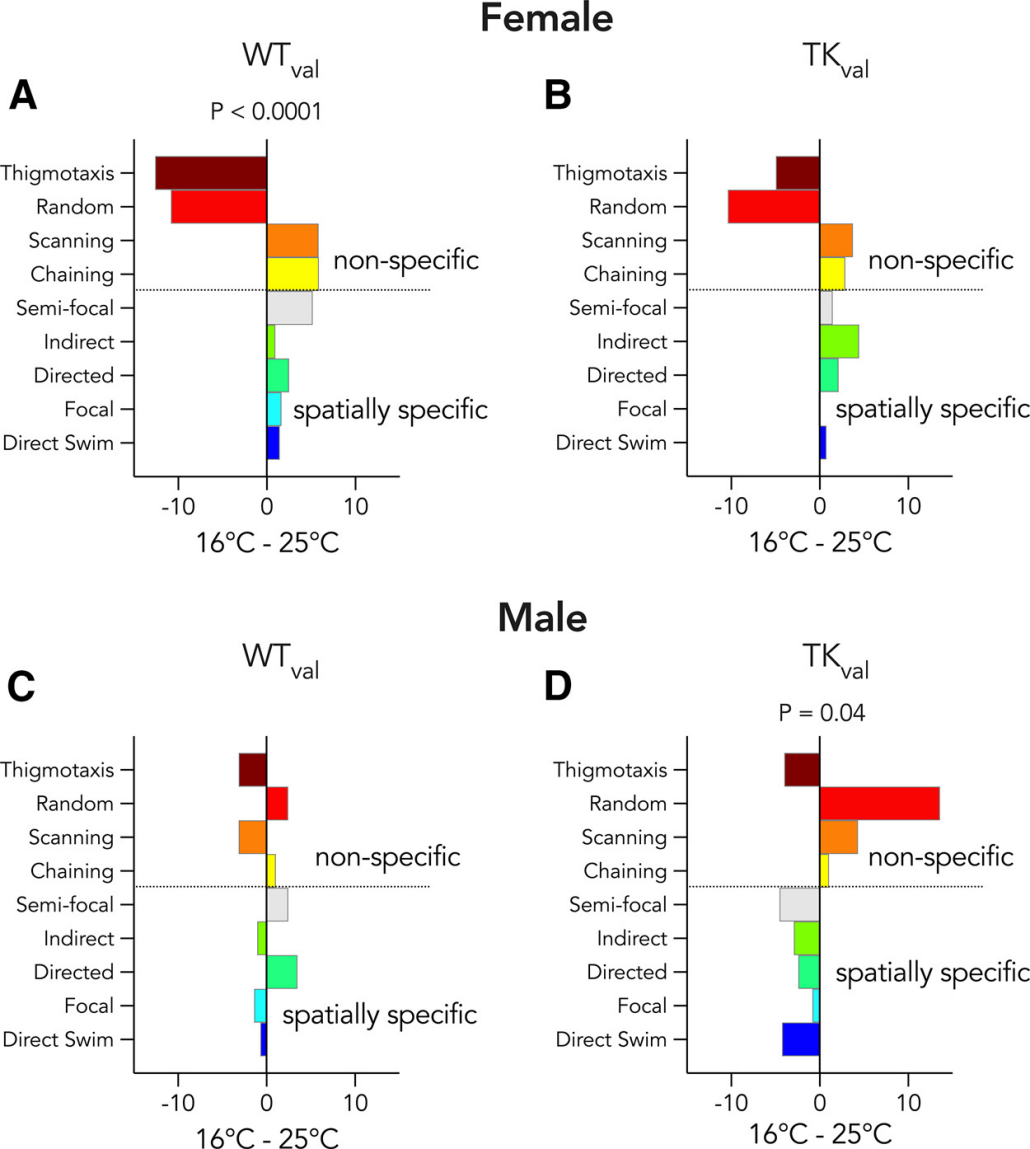
Temperature-related changes in search strategy. Graphs show difference scores for valganciclovir-treated rats trained at 16°C versus 25°C. ***A***, WT females employed different strategies as a function of temperature, and performed fewer thigmotactic and random searches at 16°C (χ^2^ = 38, *p* < 0.0001, V = V = 0.99). ***B***, TK females’ strategy did not differ across temperatures (χ^2^ = 14, *p* = 0.36, V = 0.62). ***C***, Male WT rats did not alter strategies as a function of temperature (χ^2^ = 9.4, *p* = 1.0, V = 0.46). ***D***, Male TK rats performed fewer spatially specific searches at 16°C (χ^2^ = 23, *p* = 0.04, V = 0.74).

**Figure 8.**
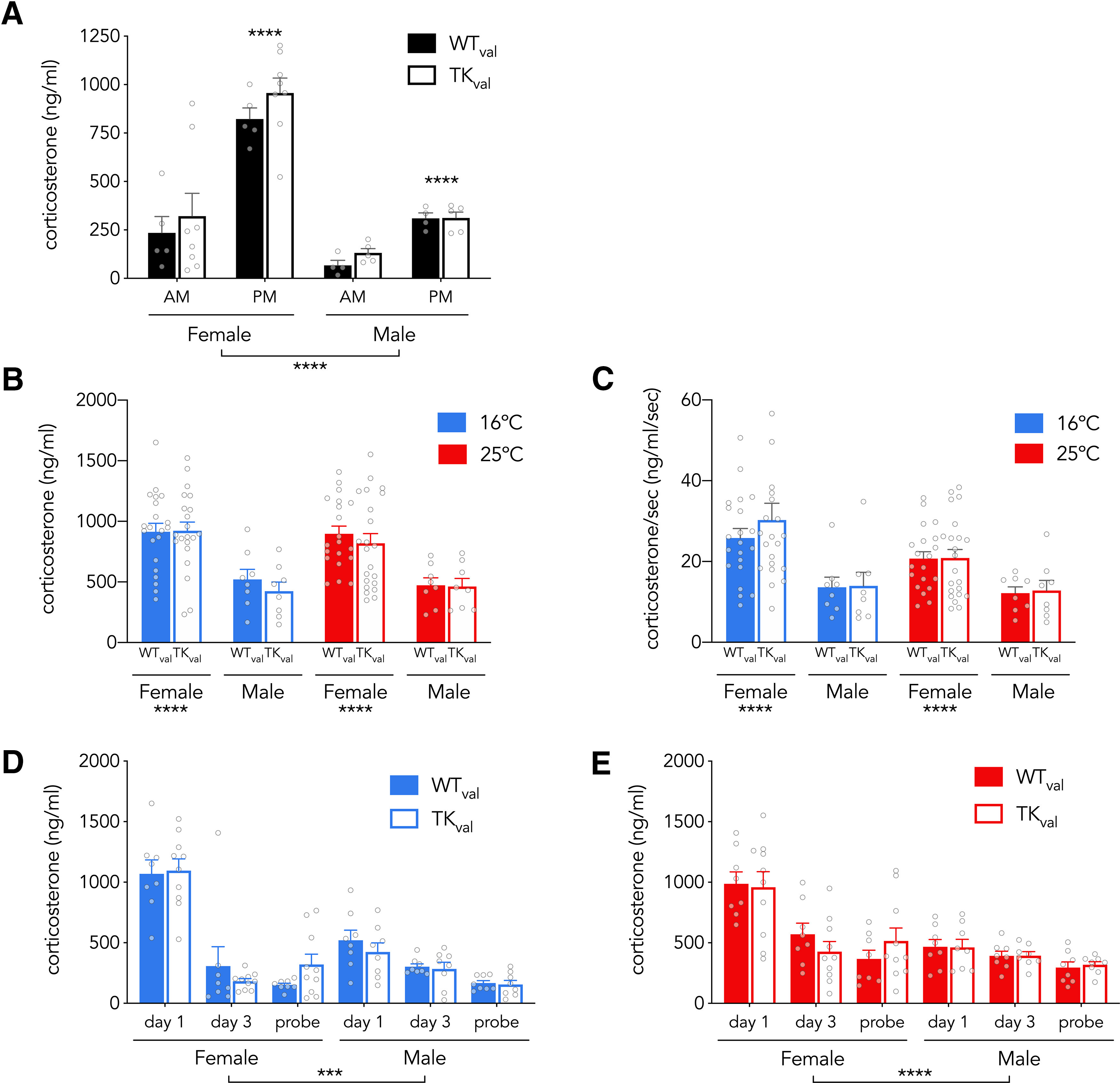
Similar HPA reactivity in valganciclovir-treated WT and TK rats. ***A***, Baseline corticosterone varies across circadian phase and sex, but not neurogenesis (effect of time of day: *F*_(1,18)_ = 55, *p* < 0.0001, η_p_^2^ = 0.74; effect of sex: *F*_(1,18)_ = 34, *p* < 0.0001, η_p_^2^ = 0.65; effect of genotype: *F*_(1,18)_ = 1.2, *p* = 0.3, η_p_^2^ = 0.06; genotype interactions all *p* > 0.5). ***B***, On day 1 of acquisition, corticosterone levels were higher in females but were not different between genotypes or between rats trained at 16°C or 25°C (effect of sex: *F*_(1,108)_ = 48, *p* < 0.0001, η_p_^2^ = 0.29; effect of genotype: *F*_(1,108)_ = 0.5, *p* = 0.5, η_p_^2^ = 0.48; effect of temperature: *F*_(1,108)_ = 0.3, *p* = 0.6, η_p_^2^ = 0; genotype interactions all *p* ≥ 0.5). ***C***, Day 1 corticosterone, normalized to time spent in the water maze, was higher in females but not significantly different between genotypes or rats trained at 16°C or 25°C (effect of sex: *F*_(1,108)_ = 22, *p* < 0.0001, η_p_^2^ = 0.17; effect of genotype: *F*_(1,108)_ = 0.3, *p* = 0.6, η_p_^2^ = 0; effect of temperature: *F*_(1,108)_ = 3.2, *p* = 0.07, η_p_^2^ = 0.03; interactions all *p* > 0.2). ***D***, HPA activity habituated over days in the 16°C water maze but did not differ between genotypes (effect of day: *F*_(2,60)_ = 96, *p* < 0.0001, η_p_^2^ = 0.76; effect of sex: *F*_(1,30)_ = 13, *p* = 0.001, η_p_^2^ = 0.30; effect of genotype: *F*_(1,30)_ = 0.0, *p* = 0.9, η_p_^2^ = 0; genotype interactions all *p* > 0.2). ***E***, HPA activity habituated over days in the 25°C water maze but did not differ between genotypes (effect of day: *F*_(2,60)_ = 26, *p* < 0.0001, η_p_^2^ = 0.46; effect of sex: *F*_(1,30)_ = 18, *p* = 0.0002, η_p_^2^ = 0.38; effect of genotype: *F*_(1,30)_ = 0.0, *p* = 1, η_p_^2^ = 0; genotype interactions all *p* > 0.3). Bars indicate mean ± SE. ***p < 0.001, ****p < 0.0001.

**Figure 9.**
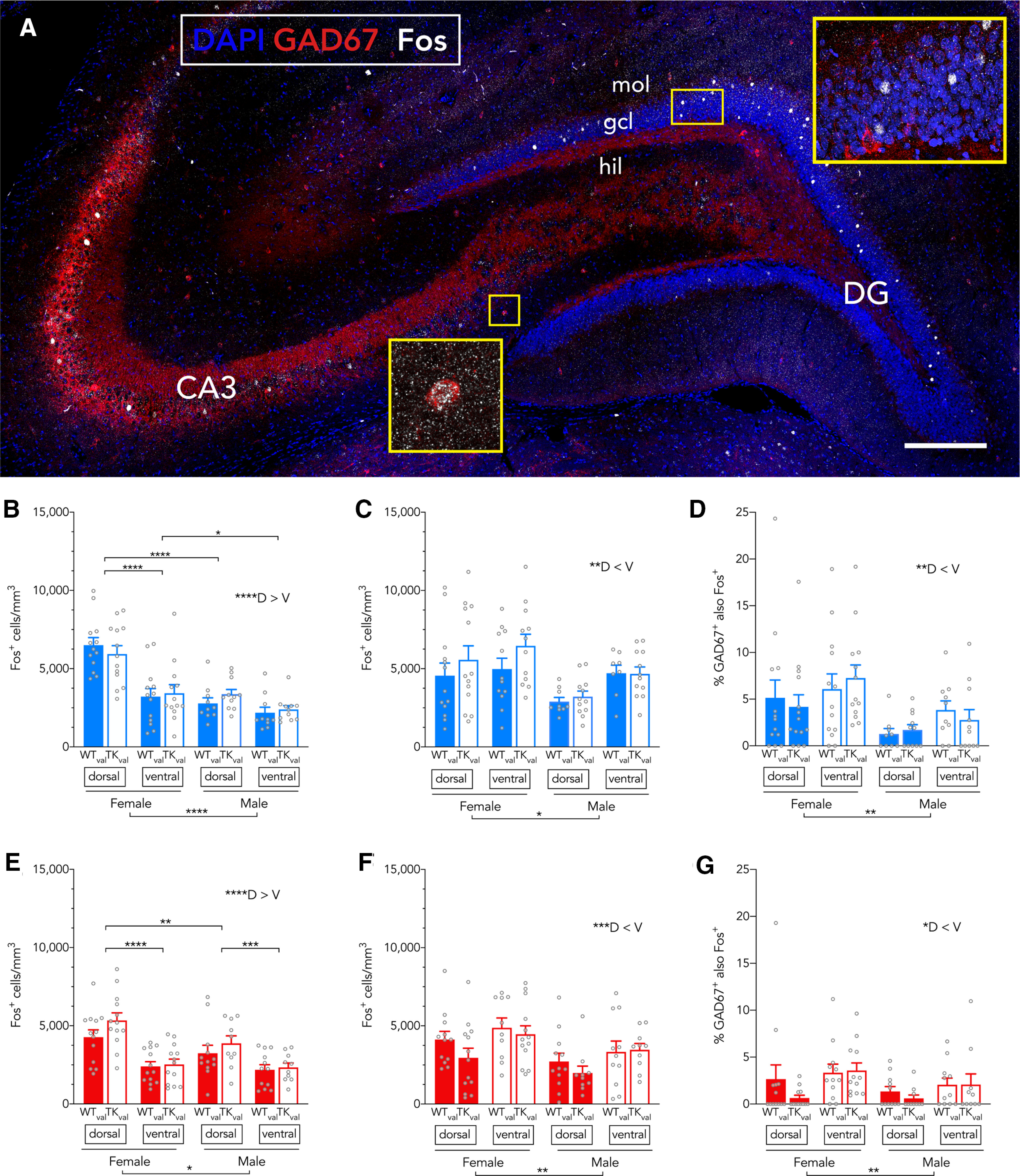
Sex-based and subregion-based activation of DG-CA3 neurons in valganciclovir-treated rats. ***A***, Confocal image of dorsal hippocampus immunostained for GAD67 and Fos. Scale bar: 200 μm. ***B***, In the 16°C water maze, there were more Fos^+^ cells in the dorsal granule cell layer, particularly in females (effect of subregion: *F*_(1,43)_ = 52, *p* < 0.0001, η_p_^2^ = 0.55; effect of sex: *F*_(1,43)_ = 31, *p* < 0.0001, η_p_^2^ = 0.42; effect of genotype: *F*_(1,43)_ = 0.1, *p* = 0.8, η_p_^2^ = 0; subregion × sex interaction: *F*_(1,43)_ = 17, *p* = 0.0002, η_p_^2^ = 0.29; all other interactions *p* > 0.25; female dorsal vs ventral: g = 1.52, male dorsal vs ventral: g = 0.75, dorsal female vs male: g = 2.00). ***C***, At 16°C, there were more Fos^+^ cells in CA3 in females and in the ventral subregion (effect of subregion: *F*_(1,40)_ = 9.2, *p* = 0.004, η_p_^2^ = 0.19; effect of sex: *F*_(1,43)_ = 6.8, *p* = 0.01, η_p_^2^ = 0.14; effect of genotype: *F*_(1,43)_ = 1.5, *p* = 0.2, η_p_^2^ = 0.03; all interactions *p* ≥ 0.16). ***D***, In the 16°C water maze, there were more GAD67^+^Fos^+^ cells in females and in the ventral granule cell layer (effect of subregion: *F*_(1,42)_ = 8, *p* = 0.006, η_p_^2^ = 0.17; effect of sex: *F*_(1,44)_ = 8, *p* = 0.009, η_p_^2^ = 0.15; effect of genotype: *F*_(1,44)_ = 0.0, *p* = 0.99, η_p_^2^ = 0; all interactions: *p* > 0.17). ***E***, In the 25°C water maze, there were more Fos^+^ cells in females and in the dorsal granule cell layer, but sex and subregion differences were modest compared with 16°C (effect of subregion: *F*_(1,44)_ = 74, *p* < 0.0001, η_p_^2^ = 0.63; effect of sex: *F*_(1,44)_ = 4.2, *p* = 0.048, η_p_^2^ = 0.09; effect of genotype: *F*_(1,44)_ = 1.9, *p* = 0.2, η_p_^2^ = 0.04; subregion × sex interaction: *F*_(1,44)_ = 6, *p* = 0.02, η_p_^2^ = 0.12; all other interactions *p* > 0.09; female dorsal vs ventral: g = 1.52; male dorsal vs ventral: g = 0.91; dorsal female vs male: g = 0.73; ventral female vs male: g = 0.19). ***F***, In the 25°C water maze, there were more Fos^+^ cells in CA3 in females and in the ventral subregion (effect of subregion: *F*_(1,40)_ = 13, *p* = 0.0009, η_p_^2^ = 0.24; effect of sex: *F*_(1,43)_ = 7.5, *p* = 0.009, η_p_^2^ = 0.15; effect of genotype: *F*_(1,43)_ = 1.4, *p* = 0.2, η_p_^2^ = 0.03; all interactions *p* > 0.22). ***G***, In the 25°C water maze, there were more GAD67^+^Fos^+^ cells in the ventral hippocampus but there were no sex or genotype differences (effect of subregion: *F*_(1,40)_ = 5.2, *p* = 0.03, η_p_^2^ = 0.12; effect of sex: *F*_(1,43)_ = 2.5, *p* = 0.12, η_p_^2^ = 0.06; effect of genotype: *F*_(1,43)_ = 0.9, *p* = 0.3, η_p_^2^ = 0.02; all interactions: *p* > 0.24. Bars indicate mean ± SEM; mol, molecular layer; gcl, granule cell layer; hil, hilus; D, dorsal; V, ventral. *p < 0.05, **p < 0.01, ***p < 0.001, ****p < 0.0001.

For experiments with neurogenesis ablation, animals were treated orally with pellets of valganciclovir (4 mg) in a 1:1 peanut butter and rodent chow mix (0.5 g). Drug pellets were given directly to each animal to ensure accurate dosing. Animals began treatment at six to seven weeks of age, and were treated twice a week (3- to 4-d interval) for six to seven weeks before behavioral testing began. Valganciclovir treatment stopped immediately before behavioral testing. Data from these rats are indicated by “val” in subscript (Figs. 1-4, 6–9). In a separate control experiment we tested for nonspecific genotype differences between WT and TK rats that did not undergo neurogenesis ablation. Here, rats received neither valganciclovir nor peanut butter and rodent chow mix (indicated by “untreated” in subscript; Fig. 5).

### Spatial water maze testing

The water maze consisted of a white circular pool (180-cm diameter), with 60-cm-high walls. The pool was filled with water to a 32-cm depth, and the water was made opaque with addition of white nontoxic liquid tempera paint (Schola). Training contexts of high-stress or moderate-stress were created by using either 16°C or 25°C water, respectively, similar to previous work (Sandi et al., 1997; Salehi et al., 2010). The pool was located in a room (∼4 × 6 m in size) with diffuse lighting, and contained extra-maze visual cues along the room’s walls and distributed within the room (desk, computer, cabinets). A circular escape platform (12-cm diameter) was placed in the NE quadrant of the pool, and was positioned 2 cm below the water surface. Rats received 3 d of acquisition training with four trials per day. Rats were tested in groups of two to three, and during daily training sessions were placed into individual holding cages filled with aspen chip bedding and paper towel.

For each trial, rats were placed into the pool at one of four possible release locations (pseudo-random order), with each release location occurring once on each day. Rats were given a maximum of 60 s to locate the escape platform, after which they were guided to the escape platform by the experimenter. Following each trial, rats remained on the escape platform for ∼10 s and were gently dried with a towel before being returned to their holding cage for the inter-trial interval (30–90 s). The rats’ trajectory was recorded with an Ethovision (Noldus) tracking system, and performance was assessed via latency to locate the escape platform and swim speed. Ideal path error (conceptually similar to cumulative search error/proximity metrics; Gallagher et al., 1993), which can detect spatial performance differences between trials that have similar latencies and distances, was calculated with Pathfinder software (Cooke et al., 2019) as follows: the distance from the platform was summed over all samples to obtain a cumulative distance metric. To control for different release locations, the cumulative distance for the optimal path was also calculated based on a direct escape path from the release location and the average swim speed. The ideal path error was then calculated by subtracting the cumulative optimal path from the cumulative actual path. On the day following acquisition training, the platform was removed from the pool and rats completed a 60-s probe trial to assess memory. Spatial memory was measured as the time spent in a 36-cm zone surrounding the former escape platform location, and the corresponding 36-cm zones in each of the nontarget quadrants. Rats were euthanized 60 min after the probe trial to capture experience-dependent Fos expression in activated neurons (see below, Immunohistochemistry).

### Search strategy analyses

Navigational search strategies employed in the water maze were detected using Pathfinder software (Cooke et al., 2019), with the following parameters: angular corridor width: 45°, chaining annulus width: 45 cm, thigmotaxis zone width: 15 cm, direct swim maximum ideal path error: 125, max heading error: 35°; focal search max distance to swim path centroid: 30, max distance to goal: 30, min distance covered: 100 cm, max distance covered: 500 cm; directed search min time in angular corridor: 70%, max distance covered: 400 cm, max ideal path error: 1500; indirect search max ideal path error: 450, max average heading error: 70°; semi-focal search max distance to swim path centroid: 60, max distance to goal: 60, min distance covered: 200 cm, max distance covered: 5000 cm; chaining min time in annulus: 70%, min quadrants visited: 4, max area of maze traversed: 40%; scanning max area of maze traversed: 20%, min area of maze traversed: 0%, max average distance to maze center: 60; thigmotaxis time in full zone: 60%, time in smaller zone: 0%, min total distance covered: 400 cm. Random search min area of maze traversed: 5%. The small number of trials that were not categorized by Pathfinder were designated as random. Probe trial analyses were conducted on truncated trials that ended when rats reached the former platform location.

### Retrovirus injections

Moloney Murine Leukemia Virus retrovirus, produced as recently described (Seib et al., 2021), was use to express eGFP in adult-born neurons. Viral titers ranged from 1 to 8 × 10^6^ colony forming units/ml. Eight-week-old male and female rats were bilaterally injected with 1 μl of retrovirus into the dorsal dentate gyrus (anteroposterior = −4.0 mm; mediolateral = ±3.0 mm; dorsoventral = −3.5 mm from bregma). Thirty days later, rats either remained in their home cage or were trained and tested for 4 d in the 16°C or 25°C water maze, as above. Rats were perfused the next day, when cells were 35 d old.

### Blood sampling and radioimmunoassays (RIAs)

In one group of rats, different from those used to generate the main behavioral data in Figures 2, 3, 6, 7, 9, blood samples were obtained 30 min following testing sessions on days 1 and 3 of acquisition training and after the probe trial on day 4. After the last trial of a training session was completed, rats remained in the testing room for 5 min, before being returned to their home-cage and colony room for the remaining 25 min. Rats were then quickly brought into the hallway adjacent to the colony room, restrained, and blood was collected via tail vein puncture. For baseline circadian measurements, home cage control rats were sampled directly from their cage without transport. Blood was left at room temperature for 30–45 min, centrifuged, and serum supernatant was collected and stored at −80°C until analyzed by RIA. RIAs were completed using a I^125^ corticosterone competitive binding assay (MP Biomedical). In a subset of these animals, body temperature was also obtained immediately following blood sampling using a rectal thermometer.

### Vaginal lavage and estrous staging

Vaginal lavages were performed on a subset of female rats within 1–6 h of completing the probe trial. Rats were gently wrapped in a towel and rotated so that the vagina was clearly visible. The vagina was then flushed with tap water using a glass transfer pipette with a smooth, curved tip. The water was then aspirated into the pipette and collected on a glass slide. The samples were left to dry for at least 24 h before being stained in cresyl-violet (0.1% for 1 min). For animals that were used in Figures 2, 3, 6, 7, lavages were performed immediately before euthanasia and perfusion, to prevent any effects of lavage on water maze behavior or experience-dependent Fos expression. Additionally, only a portion of the animals that were used for these figures were lavaged. For animals that were used for corticosterone measurements, lavage was performed at the same time blood was collected. Identification of estrous cycle stage was completed based on the cytology of lavages, as described (McLean et al., 2012), using an Olympus CX41 light microscope. Briefly, proestrus was identified based on the presence of round squamish cells with visible nuclei, estrous with cornified squamish cells without visible nuclei, metestrus with both cornified squamish cells and leukocytes and diestrus with squamish cells that have visible nuclei and leukocytes.

### Immunohistochemistry

Animals were euthanized via overdose of isoflurane, and transcardially perfused with 4% paraformaldehyde in 0.1 m PBS (pH 7.4). Brains were dissected and incubated in 4% paraformaldehyde for an additional 24 h, after which they were placed in PBS with 0.1% sodium azide, and stored at 4°C. Before sectioning, brains were cryoprotected by incubation in 10% glycerol in PBS for 24 h, followed by 20% glycerol for 48 h. Brains were sectioned coronally through the hippocampus at 40-μm thickness using a freezing microtome and stored in cryoprotectant solution at −20°C until immunohistochemistry was completed.

For immunolabelling of doublecortin (DCX), one dorsal and one ventral section from each animal was mounted onto slides (Fisher, Superfrost) and left to dry for 24 h. Slides were incubated in 0.1 m citric acid and heated to an intermittent boil for 10 min for antigen-retrieval. Sections were then washed and incubated in PBS with 0.5% Triton X-100 and 3% horse serum for 20 min. Tissue was then incubated in PBS with Triton X-100, with mouse-anti DCX monoclonal antibody (Santa Cruz Biotechnology, sc-271390, 1:100) at 4°C for 3 d. Sections were then rinsed in PBS and incubated in biotinylated goat anti-mouse secondary antibody (Sigma, B0529,1:200) for 1 h. Sections were washed and treated with hydrogen peroxide (0.3%) in PBS for 30 min. Immunostaining was visualized through incubation in avidin-biotin-horseradish peroxidase (Vector Laboratories) for 30 min, and subsequent treatment with cobalt-enhanced 3,3′-diaminobenzidine chromogen (Sigma Fast Tablets, Sigma). Sections were then counter-stained with cresyl-violet (0.1%), dehydrated, cleared with citrisolv (ThermoFisher) and coverslipped with permount (Fisher).

For immunostaining of GFP, serial sections were incubated in mouse anti-GFP (DSHB, GFP-12E6, 1:100 in PBS with Triton X-100) for 24 h, washed, incubated for 2 h with donkey anti-mouse Alexa Fluor 488 secondary antibody, washed, mounted onto slides, and coverlipped with PVA-DABCO.

For immunostaining of c-Fos, sections were incubated in goat anti-c-Fos primary antibody (1:2000, Santa Cruz sc-52-G) in PBS-TX with horse serum for 3 d at 4°C. Sections were then washed three times in PBS-TX and then incubated in secondary biotinylated donkey anti-goat antibody (1:250, Jackson ImmunoResearch; 705-065-147) for 1 h in PBS-TX with horse serum. The sections were then washed three times in PBS-TX, incubated in blocking solution (0.5%, PerkinElmer; FP1020) for 30 min, before application of streptavidin-HRP (1:100, NEL750) for 1 h. Sections were then washed (3 × 5 min) in PBS-TX, and incubated in rhodamine (1:2000, Fisher Scientific; PI-46406) in PBS-TX and H_2_O_2_ (1:20,000) for 1 h. Sections were then washed (3 × 5 min) in PBS-TX, blocked for 30 min in PBS-TX with horse serum, and then incubated in mouse anti-GAD67 primary antibody (1:1000, Millipore MAB5406) in PBS-TX with horse serum for 3 d at 4°C. Following GAD67 antibody incubation, sections were then washed three times in PBS-TX and incubated in donkey anti-mouse Alexa Fluor 647 antibody (1:250, Invitrogen A-31571) for 1 h. Tissue was then washed in PBS-TX (3 × 5 min), and incubated in DAPI (1:1000) for 10 min. Lastly, sections were washed for (3 × 5 min) in PBS, mounted onto glass slides, and coverslipped using PVA-Dabco mounting medium.

### Quantification of immunolabelling

Quantification of all immunolabelling was completed by an experimenter blind to the experimental conditions. For DCX, the number of immuno-positive cells was counted within the granule cell layer of the DG, using an Olympus CX41 bright-field microscope with a 40× objective. The number of immuno-positive cells were counted from one section of the septal/dorsal hippocampus (bregma, −2.92 to −4.0 mm). Counts of DCX cells were also obtained from hipp3ocampal sections which contained temporal/ventral hippocampus, although counts were not separated between the suprapyramidal and infrapyramidal blades (bregma, −5.76 to −6.2 mm). Intermediate and ventral DG was delineated at 4.5 mm relative to the interaural line. All counts of DCX-positive cells were converted into densities based on the volume of the DG subregions.

For quantification of Fos immunoreactivity, a confocal microscope (Leica, SP8) was used to obtain representative z-stacks (40× objective), through the entire infrapyramidal and suprapyramidal blades of the DG, the medial and lateral blades of the ventral DG, and dorsal and ventral CA3. For each animal, an entire dorsal and ventral section was analyzed. Cells were counted as Fos-positive when the intensity of immunolabelling was more than twice that of neighboring, non-nuclei-containing, tissue in the hilus. To determine the percentage of GAD cells that also expressed Fos, Gad immune-positive cells were examined throughout the entire DG-CA3 and the proportion that expressed Fos at twice background levels was quantified.

Analyses of dendritic spine density were performed from z-stack images acquired with a 63× glycerol-immersion objective (NA 1.3). Images were 1024 × 1024 pixels in size, taken at 5× zoom, a speed of 400 Hz, and a z-height of 0.5 μm. For each neuron, images were acquired from the outer molecular layer (where lateral perforant path axons terminate), middle molecular layer (where medial perforant path axons terminate), and inner molecular layer (where mossy cell/commissural fibers terminate). All protrusions were counted as spines and mushroom spines were defined as having a head diameter ≥0.6 μm. A total of 14–37 cells per group, distributed equally across three to five animals per group, were analyzed.

Analyses of mossy fiber terminals were performed from z-stack images acquired with a 40× oil-immersion objective (NA 1.3). Images were 1024 × 1024 pixels in size, taken at 2× zoom, a speed of 400 Hz, and a z-height of 0.5 μm. The area of the large mossy terminal was measured from maximum intensity projections and the number of terminal-associated filopodia, >1 μm in length, was also quantified as a proxy for GABAergic interneuron innervation (Acsády et al., 1998; Restivo et al., 2015). Large mossy terminals and filopodia were categorized according to their position along the proximodistal CA3 axis, where CA3a is the curved distal portion of CA3, CA3c is proximal and enclosed within the blades of the DG, and CA3b is the intermediate CA3 region. A total of 59–122 large mossy terminals per group, distributed equally across three to five animals per group, were analyzed.

### Statistical analyses

Analyses of water maze acquisition performance were performed using mixed-design repeated measures ANOVA with sex and genotype as between-subject factors and training day as a within subject factor. Valganciclovir-treated rats and untreated rats were tested at different times and therefore were analyzed separately to reduce the risk of type-2 errors caused by variability associated with baseline differences in behavior. The distribution of search strategies in WT and TK rats was analyzed by a χ^2^ test with Bonferroni correction for multiple comparisons. Probe trial performance was analyzed with between-subject ANOVAs (sex × genotype). For behavioral experiments, 16°C and 25°C groups were typically analyzed and presented separately; in some cases, we directly compared 16°C and 25°C groups to explore temperature effects. Since our primary objective was to examine neurogenesis effects within the sexes, and since previous studies have investigated general sex differences in water maze behavior, behavioral data are largely segregated by sex, although key sex comparisons are also highlighted. Cell densities were analyzed by mixed-design repeated measures ANOVA, or mixed effects models, with sex and genotype as between subject factors and dorsoventral subregion as a within-subjects factor. Neuronal morphology (spines, boutons, filopodia) was analyzed by ANOVA with sex and treatment as between-subjects factors. Analyses were performed with GraphPad Prism software and effect sizes were calculated using the spreadsheet provided by Lakens (2013) and the MOTE effect size calculator (https://doomlab.shinyapps.io/mote/). In all cases, where significant interactions were detected, *post hoc* comparisons were analyzed with Sidak tests. The significance level, α, was set at 0.05 for all tests.

In most cases, statistical results are presented in the figure legends alongside their respective data; for data that is not presented in figures, statistical results are presented in the results text. All statistical results and the full datasets are also available as extended material. Three rats (one female TK_val_ and two female TK_untreated_) were excluded from the study because they failed to learn (average escape latencies across the 12 trials: 60, 60, 52 s) and probe trial data were not acquired for two rats because of technical errors (25°C condition: female WT_val_ and female TK_val_).

## Results

Detailed statistical analyses, and the full dataset underlying all analyses, can be found in Extended Data Figure 1-1.

10.1523/ENEURO.0054-22.2022.f1-1 Extended Data Figure 1-1 Excel spreadsheet containing the complete statistical analyses as well as the datasets underlying each figure. Download Figure 1-1, XLS file.

### Inhibition of neurogenesis in male and female TK rats

To establish that neurogenesis was effectively inhibited along the dorsoventral axis of the DG in both male and female TK rats, we quantified the density of cells expressing the immature neuronal marker, DCX. As expected, in WT rats DCX^+^ cells were observed at the border of the granule cell layer and the hilus, in the subgranular zone (Fig. 1*A*). DCX^+^ cell density was dramatically reduced in both male and female TK rats, to <15% of levels found in WT littermates, comparable to previous studies (Snyder et al., 2016; Seib et al., 2018, 2021). This reduction was observed in the dorsal and ventral hippocampus, and there were no sex differences in the extent of neurogenesis reduction (Fig. 1*B*).

### In cold water, ablation of neurogenesis impairs spatial learning in male rats and improves spatial learning in female rats

Ablating neurogenesis typically does not impair learning a single spatial location in the water maze (Shors et al., 2002; Madsen et al., 2003; Raber et al., 2004; Snyder et al., 2005; Saxe et al., 2006; Jessberger et al., 2009; Blaiss et al., 2011; Groves et al., 2013; Nickell et al., 2020). Since adult-born neurons regulate unconditioned responses to stressors (Snyder et al., 2011; Seo et al., 2015; Schoenfeld et al., 2021), we hypothesized that stress or aversiveness may also reveal a role for new neurons in spatial learning. We therefore tested WT and TK rats in the spatial water maze at standard temperatures (25°C) or colder, more aversive temperatures (16°C).

In the 16°C water maze, blocking neurogenesis altered learning in both males and females, but in opposite directions: male TK rats located the platform slower but female TK rats located it faster, compared with their WT counterparts (Fig. 2*A–C*). WT male rats located the platform faster than WT females. On the probe trial, TK male rats tended to spend less time searching in the target zone but this difference was not statistically significant (Fig. 2*D–F*). We explored whether estrous stages influenced probe trial performance (but not training, to avoid lavage impacts on subsequent behavior). Following the 16°C probe trial, the estrous distribution of female WT and TK rats did not differ (χ^2^ = 2.7, *p* = 0.4) and there was no effect of estrous stage on probe trial performance (Fig. 2*G*).

In standard 25°C water, WT and TK rats learned to escape from the pool with similar latencies (Fig. 2*H–J*) and, in the probe trial, WT and TK rats displayed equivalent memory (Fig. 2*K–M*). We observed sex differences in performance, where males escaped faster and spent more time in the target zone than females. The distribution of WT and TK rats across the four stages of the estrous cycle did not differ (χ^2^ = 1.3, *p* = 0.7) but rats in proestrus displayed better memory, similar to what has been observed during training in warm water (Rubinow et al., 2004; Fig. 2*N*).

Neurogenesis-related differences in escape latency were not because of differences in swim speed (Fig. 3*A–D*). A similar pattern of sex and genotype differences was observed when we analyzed ideal path error, a measure of the cumulative positional error relative to the platform that is not influenced by differences in swim speed or path length (Cooke et al., 2019): at 16°C female TK rats had a lower path error and male TK rats had a greater path error, relative to WT controls. At 25°C, WT and TK rats did not differ (Fig. 3*E–J*).

To rule out the possibility that behavioral differences were because of nonspecific physiological effects caused by cold water, we measured body temperature in a separate group of rats. At both 16°C and 25°C, body temperature was lowest after day 1 training, was lower on day 1 in females than in males, but not different between WT and TK rats (Fig. 4). Male TK rats weighed slightly less than male WT rats, consistent with previous studies showing that neurogenesis inhibition can sometimes reduce weight (Snyder et al., 2005, 2016; 8%; WT: 480 ± 8 g, TK: 441 ± 8 g; mean ± SEM). However, female WT and TK rats were not different (3%; WT: 279 ± 5 g, TK: 270 ± 6 g; two-way ANOVA; effect of genotype: *F*_(1,116)_ = 11, *p* = 0.001; genotype × sex interaction: *F*_(1,116)_ = 4, *p* = 0.049; female WT vs TK: *p* = 0.6; male WT vs TK: *p* = 0.0001). Furthermore, neither body weight nor body temperature correlated with learning and memory performance at 16°C or 25°C, suggesting that water temperature did not differentially impact sexes or genotypes because of hypothermic effects (Tables 1, 2). Finally, to rule out the possibility that TK impairments and enhancements in learning are because of nonspecific effects of the GFAP-TK transgene, we trained additional WT and TK rats that did not receive valganciclovir treatment. Here, no genotype differences were observed at 16°C or 25°C water temperatures (Fig. 5).

**Table 1:** Correlations between body weight and learning and memory

	Day 1 latency	Day 2 latency	Day 3 latency	Probe
16°C males (*N* = 36)	0.13 (0.45)	−0.19 (0.26)	−0.21 (0.22)	0.08 (0.63)
16°C females (*N* = 24)	−0.05 (0.81)	−0.23 (0.28)	−0.13 (0.54)	0.16 (0.45)
25°C males (*N* = 36)	−0.04 (0.81)	0.24 (0.16)	−0.24 (0.15)	−0.10 (0.56)
25°C females (*N* = 24)	0.23 (0.27)	0.22 (0.28)	0.06 (0.77)	−0.32 (0.15)

Pearson *r* correlation coefficients and uncorrected *p* values in brackets. Body weight was not significantly correlated with performance on the water maze (Bonferroni-corrected α = 0.0031).

**Table 2:** Correlations between body temperature and learning and memory

	Day 1 latency	Day 3 latency	Probe
16°C males (*N* = 16)	−0.32 (0.23)	−0.45 (0.08)	0.06 (0.63)
16°C females (*N* = 18)	−0.34 (0.17)	−0.30 (0.22)	0.19 (0.44)
25°C males (*N* = 16)	−0.07 (0.79)	−0.11 (0.70)	−0.14 (0.62)
25°C females (*N* = 18)	−0.58 (0.02)	−0.48 (0.05)	0.20 (0.44)

Pearson *r* correlation coefficients and uncorrected *p* values in brackets. Body temperature was not significantly correlated with performance on the water maze (Bonferroni-corrected α = 0.0042).

To gain insight into navigational strategies employed during learning, we analyzed search patterns with Pathfinder software (Cooke et al., 2019). Generally, rats displayed increasing use of spatially-specific search strategies over days of testing (Fig. 6). Specifically, they shifted from thigmotaxic and random searches, or searches that covered multiple areas of the pool equally, to searches that were biased toward the escape platform with increasing precision. Male TK rats relied less on spatially-specific search strategies than their WT counterparts. Consistent with their faster escape latency, female TK rats tended to display more spatially-specific searches than their WT counterparts but this difference was not statistically significant. Consistent with the latency and path error data, search strategies did not differ between WT and TK rats tested at 25°C.

Behavioral sex differences often reflect differences in strategy (Brake and Lacasse, 2018; Shansky, 2018). We therefore explored whether maze aversiveness caused males and females to employ different navigational strategies in the water maze. Female WT rats responded strongly to cold temperature, and spent less time searching randomly and at the edge of the pool, and more time performing spatial searches in the center of the pool and near the platform. Temperature-dependent changes in search strategy were absent in female TK rats that lacked neurogenesis (Fig. 7). In contrast, male WT rats employed similar strategies at both 16°C and 25°C, but blocking neurogenesis led to temperature-dependent differences, where TK males performed fewer spatially precise searches in 16°C water. Thus, neurogenesis promoted aversiveness-related changes in search strategy in females but it promoted consistent search strategies in males.

### Blocking neurogenesis did not alter the HPA response

Neurogenesis regulates the HPA axis in mice (Snyder et al., 2011) and cold temperatures can enhance water maze learning via glucocorticoid-dependent mechanisms (Sandi et al., 1997). We therefore explored whether neurogenesis regulates HPA axis function in rats at baseline and after learning. Consistent with previous work in mice (Snyder et al., 2011), we found no neurogenesis-related changes in baseline circadian HPA function. Corticosterone levels were highest at the onset of darkness, they were higher in females, but they did not differ between WT and TK rats (Fig. 8). When corticosterone was measured 30 min after the first day of acquisition training, both WT and TK rats displayed high levels of corticosterone, which did not differ between genotypes. Corticosterone levels also did not differ between rats trained at 16°C versus 25°C. When normalized to escape latency, i.e., time spent in the water, there was a tendency for greater corticosterone levels at 16°C but this did not reach statistical significance. A subset of rats that were subjected to the full 4 d of testing displayed HPA habituation, but no corticosterone differences were observed between genotypes or temperatures. Thus, females elicit a stronger HPA response than males, but neurogenesis-associated behavioral differences at 16°C are not because of differences in HPA output.

### Activity-induced Fos expression varies by sex and dorsoventral location but is not modulated by immature neurons

Behaviorally-relevant DG neuronal populations express the activity-dependent immediate-early gene, c-Fos (Snyder et al., 2009; Liu et al., 2012; Erwin et al., 2020). To determine whether blocking neurogenesis alters neuronal population activity in males and females, we quantified Fos expression in excitatory principal cell populations in DG-CA3, in both WT and TK rats (Fig. 9). Notably, Fos activation was never different between WT and TK rats. However, more dentate granule neurons were active in females than in males, particularly at 16°C (74% more at 16°C, 24% more at 25°C). There were also strong dorsoventral gradients of activity: at 16°C, females had ∼2× greater Fos levels in the dorsal DG compared with the ventral DG or the dorsal DG of males. In contrast, males trained at 16°C did not display a significant dorsoventral gradient of activity. At 25°C, females also displayed a strong dorsoventral gradient of activity but in males this effect was weaker with only TK rats having significantly greater Fos activation in the dorsal DG. To explore whether Fos levels differed across training temperatures, we pooled genotypes and performed a sex × temperature ANOVA (dorsal and ventral subregions combined). A significant interaction revealed that females had more Fos^+^ cells when trained at 16°C than at 25°C; males did not differ (effect of sex: *F*_(1,91)_ = 30, *p* < 0.0001, effect of temperature: *F*_(1,91)_ = 3.3, *p* = 0.07; interaction *F*_(1,91)_ = 6.8, *p* = 0.01; female 16°C vs 25°C: *p* = 0.008; male 16°C vs 25°C: *p* = 0.95).

Since adult-born neurons can influence DG-CA3 activity via efferent connections with inhibitory interneurons (Restivo et al., 2015; Drew et al., 2016), we quantified Fos^+^ inhibitory, GAD67-expressing neurons in DG-CA3 (Fig. 9*D*,*G*). Generally, Fos expression was weaker in GAD67^+^ cells than in excitatory principal neurons of the DG and CA3, but clear differences could be detected by objective quantification (of cells that expressed Fos at 2× background). In rats trained at 16°C, there was a strong dorsoventral gradient of activity in GAD67^+^ cells, with greater activity in the ventral DG than in the dorsal DG. There was also significantly greater activation of GAD67^+^ cells in females than in males, but no differences because of loss of adult neurogenesis. At 25°C, fewer GAD67^+^ cells were activated (mixed effects analysis; effect of temperature: *F*_(1,91)_ = 8.2, *p* = 0.005) and the dorsoventral gradient (V > D) was weaker. In contrast to rats trained at 16°C, there were no sex differences in activation of GAD67^+^ cells in rats trained at 25°C. Finally, at 25°C there also were no differences between genotypes. Fos expression in the CA3 pyramidal cell layer was also greater in females, and greater in the ventral hippocampus, but no differences were observed in WT versus TK rats. Since a shift in reliance on the ventral-to-dorsal hippocampus mediates the progression toward spatially-specific search strategies (Ruediger et al., 2012), we explored relationships between Fos activation of dorsal versus ventral hippocampus with performance on the acquisition and retrieval stages of testing, however, no significant correlations were observed (data not shown).

### Training-dependent and sex-dependent morphologic plasticity in adult-born neurons

**Figure 10.**
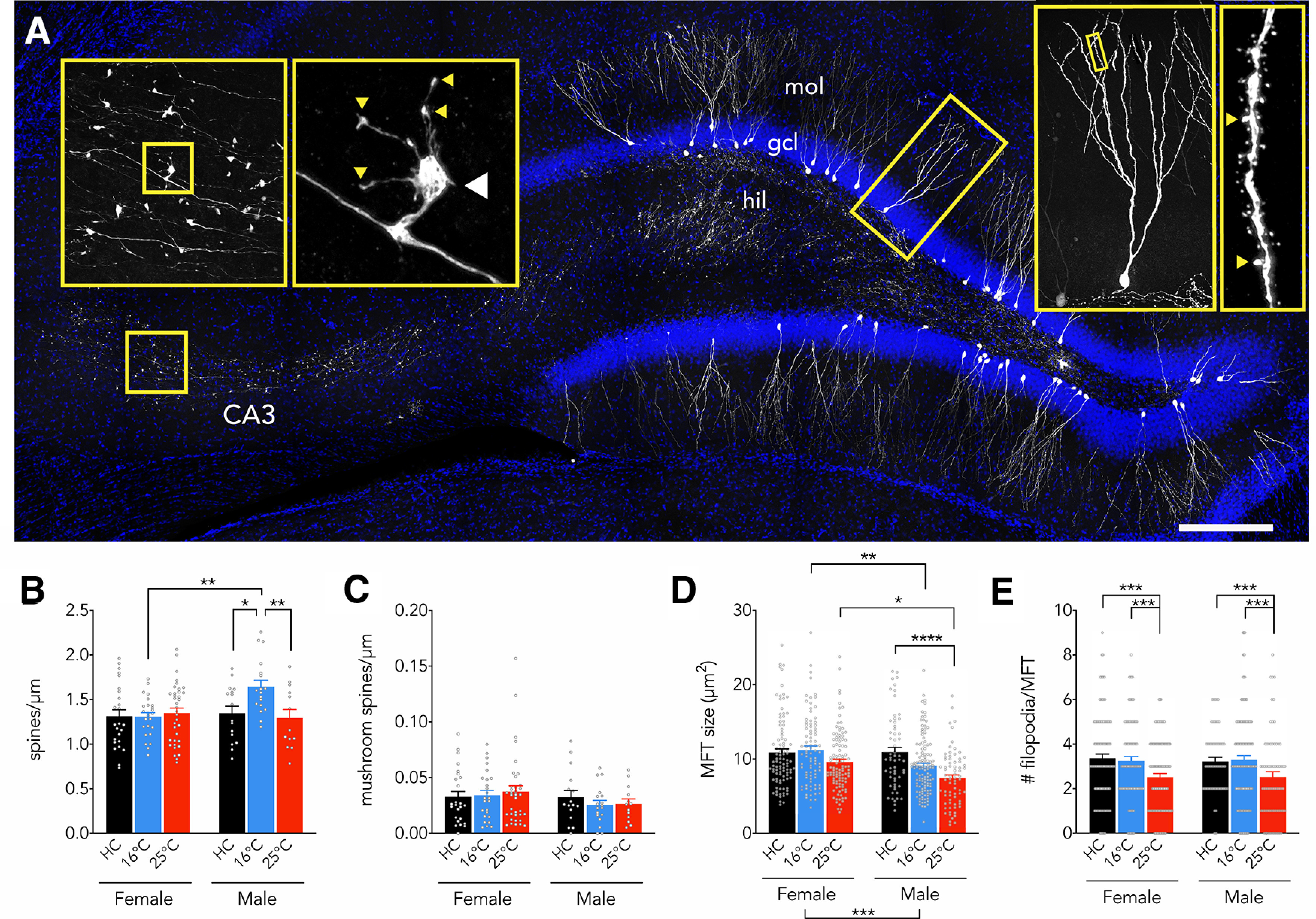
Water maze-induced morphologic plasticity. ***A***, Retroviral GFP labeling of adult-born neurons in the dentate gyrus, with axons projecting to CA3. Right insets display an isolated neuron (reconstructed across sections, hence the greater number of dendrites) and dendrite (arrowheads indicate mushroom spines). Left insets display a large mossy fiber terminal (MFT); white arrowhead indicates the MFT and yellow arrowheads indicate putative presynaptic filopodial contacts onto inhibitory interneurons. Scale bar: 250 μm. hil, hilus; gcl, granule cell layer; mol, molecular layer. ***B***, Adult-born neuron spine density was selectively increased in male rats that were trained at 16°C [effect of treatment: *F*_(2,127)_ = 3.1, *p* = 0.0495, η_p_^2^ = 0.046; effect of sex: *F*_(1,127)_ = 3.2, *p* = 0.08, η_p_^2^ = 0.076; interaction: *F*_(2,127)_ = 4.2, *p* = 0.02, η_p_^2^ = 0.06; male home cage (HC) vs 16°C: *p* = 0.01, g = 0.93; male HC vs 25°C: *p* = 0.7, g = 0.16; male 16°C vs 25°C: *p* = 0.009, g = 1.05; female group comparisons all *p* > 0.95; male vs female at 16°C: *p* = 0.003, g = 1.26; male vs female HC and male vs female at 25°C both *p* > 0.8]. ***C***, Adult-born neuron mushroom spine density was not altered by sex or training (effect of treatment: *F*_(2,127)_ = 0.88, *p* = 0.12, η_p_^2^ = 0; effect of sex: *F*_(1,127)_ = 2.0, *p* = 0.15, η_p_^2^ = 0.02; interaction: *F*_(2,127)_ = 0.5, *p* = 0.6, η_p_^2^ = 0). ***D***, MFTs were larger in adult-born neurons from female rats, an effect that was driven by greater training-related reduction in terminal size in males (effect of sex, *F*_(1,539)_ = 14, *p* = 0.0002, η_p_^2^ = 0.025; effect of training condition *F*_(2,539)_ = 13, *p* < 0.0001, η_p_^2^ = 0.047; interaction, *F*_(2,539)_ = 3.5, *p* = 0.03, η_p_^2^ = 0.013; male HC vs male 16°C, *p* = 0.07, g = 0.43; male HC vs male 25°C, *p* < 0.0001, g = 0.80; female HC vs female 16°C and 25°C both *p* > 0.18 and g < 0.3; male HC vs female HC, *p* = 0.9, g = 0.01; male 16°C vs female 16°C, *p* = 0.005, g = 0.51; male 25°C vs female 25°C, *p* = 0.01, g = 0.56). ***E***, The number of MFT-associated filopodia, putative synapses onto inhibitory neurons, was reduced in the 25°C group but was not different between sexes (effect of training condition, *F*_(2,545)_ = 9, *p* < 0.0001, η_p_^2^ = 0.033, effect of sex, *F*_(1,545)_ = 0, *p* = 0.9, η_p_^2^ = 0; interaction, *F*_(2,545)_ = 0.1, *p* = 0.9, η_p_^2^ = 0). Bars indicate mean ± SEM mol, molecular layer; gcl, granule cell layer; hil, hilus. *p < 0.05, **p < 0.01, ***p < 0.001, ****p < 0.0001.

Functionally-relevant morphologic features of adult-born neurons develop during the weeks and months postmitosis (Zhao et al., 2006; Gonçalves et al., 2016; Cole et al., 2020) and can be modified by spatial learning (Tronel et al., 2010; Lemaire et al., 2012). To examine sex differences in experience-dependent plasticity, we labeled adult-born neurons with retrovirus and analyzed GFP^+^ spines and presynaptic terminals as morphologic proxies for afferent and efferent connectivity (Fig. 10). At baseline, in naive home cage rats, there were no differences in spine density between adult-born neurons from male and female rats. However, in male rats, training at 16°C elevated spine density compared with rats that were untrained or trained at 25°C, and compared with female rats trained at 16°C. This effect was observed throughout the molecular layer (treatment × subregion interaction: *F*_(4,88)_ = 2.1, *p* = 0.09). In both males and females, regardless of treatment, spine density increased with distance from the cell soma (not shown) as described previously (Cole et al., 2020). The density of large, mushroom spines was not altered by training (Fig. 10*C*).

Finally, we examined the large mossy fiber terminals that excite CA3 pyramidal neurons. No sex differences were observed between naive, home cage control rats. However, only in males, training decreased mossy fiber terminal size, an effect that was greatest in the 25°C group (Fig. 10*D*). In both females and males, 25°C training also reduced the number of filopodial extensions, the morphologic sites of synapses onto inhibitory neurons, that protrude off of mossy fiber boutons (Acsády et al., 1998; Fig. 10*E*).

## Discussion

There are sex differences in hippocampal memory, plasticity and physiology (Koss and Frick, 2017). And while there is also evidence that the addition and activation of new neurons differs between males and females (Yagi and Galea, 2019), few studies have formally investigated sex differences in neurogenesis, especially in animals that have altered adult neurogenesis (Knudson et al., 2022). Here, we report that blocking neurogenesis caused female rats to escape faster and male rats to escape slower, relative to intact rats in a spatial water maze at aversive 16°C temperatures. Neurogenesis effects on acquisition latency were not because of genotype differences in swim speed, body weight, or body temperature. They were also validated by analyses of rats’ deviation from an ideal path to the target (ideal path error), a measure of spatial accuracy that is not confounded by differences in swim speed or path length (Cooke et al., 2019). Finally, behavioral changes were not present in TK rats that were not treated with valganciclovir (and therefore had intact neurogenesis). It is worth noting that TK rats also have reduced neurogenesis in the subventricular zone-olfactory bulb (Snyder et al., 2016). However, given the critical role of the hippocampus in spatial learning, the most likely explanation for our behavioral results is the loss of newborn neurons in the dentate gyrus. Whereas new neurons were morphologically equivalent at baseline, water maze training evoked distinct patterns of presynaptic and postsynaptic plasticity depending on sex. Our study therefore provides new evidence that adult-born neurons make unique sex-dependent contributions to spatial learning under stress and have distinct plasticity profiles in male and female rats.

### Temperature-dependent spatial functions of newborn neurons

While some have reported acquisition and short term reference memory deficits in the spatial water maze in neurogenesis-deficient animals (Dupret et al., 2008; Garthe et al., 2009; Lemaire et al., 2012), a majority of studies have found intact spatial learning (Shors et al., 2002; Madsen et al., 2003; Raber et al., 2004; Snyder et al., 2005; Saxe et al., 2006; Jessberger et al., 2009; Blaiss et al., 2011; Groves et al., 2013; Yu et al., 2019; Nickell et al., 2020), raising questions about the necessity of adult neurogenesis for spatial learning. Our findings indicate that the degree of stress and/or aversiveness present at the time of learning is critical (as suggested by Dranovsky and Leonardo, 2012). Indeed, there is ample evidence that neurogenesis regulates innate fear and anxiety-like behaviors in response to stressful and aversive stimuli (Revest et al., 2009; Lagace et al., 2010; Snyder et al., 2011; Lehmann et al., 2013; Anacker et al., 2018; Schoenfeld et al., 2019). And while stress is known to potently modulate hippocampal memory, few studies have examined a role for neurogenesis in learning as a function of stress: one study found that neurogenesis is critical for context fear memory when mice receive a single, but not multiple, footshocks (Drew et al., 2010); another found that TK rats made more errors in a dry spatial maze only when an aversive odor was present (Schoenfeld et al., 2021).

Cold water is known to activate the DG/hippocampus (Bilang-Bleuel et al., 2005; Bohacek et al., 2015), and it can promote water maze learning in a glucocorticoid-dependent fashion (Sandi et al., 1997; Akirav et al., 2004). However, as reported elsewhere (Rubinow et al., 2004), we did not find significant differences in HPA activation between rats trained at 16°C and 25°C. While this does not rule out a role for glucocorticoids (e.g., they could act on newborn neurons; Fitzsimons et al., 2013), it raises the question of whether 16°C water is indeed more stressful than 25°C water. One possibility is that stress-related differences were masked by ceiling effects on the HPA response, at least on day 1 of training. Alternatively, 16°C water stress may have influenced performance through noradrenergic (McIntyre et al., 2012) or dopaminergic (Tsetsenis et al., 2021) mechanisms. In any case, 16°C water evoked physiological changes and behaviors that are broadly consistent with the concept of a stressor as a stimulus that perturbs an organism from baseline and induces an adaptive or homeostatic response (Ulrich-Lai and Herman, 2009).

### Sex differences in the behavioral function of adult-born neurons

We found that blocking neurogenesis led to opposite behavioral outcomes in females and males. To date, sex differences in function have gone largely undetected because few studies have compared male and female animals that have altered neurogenesis. In our recent analysis of the literature (Knudson et al., 2022), we counted only four functional studies that have reported data by sex or included sex as a variable in their statistical analyses (Huckleberry et al., 2018; Seib et al., 2018; Miller et al., 2019; Cope et al., 2020). Very recently, two studies have identified functional differences in male and female rodents that lack adult neurogenesis. One study found sex differences in the circadian HPA response of GFAP-TK rats (Silveira‐Rosa et al., 2021), which would appear to conflict with our finding that baseline HPA output is not different in rats that lack neurogenesis. This discrepancy could be because of methodological differences, including potential strain-related, neurogenesis-independent alterations in stress and emotional regulation (Groves et al., 2013). Another study found that blocking adult neurogenesis selectively reduced anxiety-like behavior in male mice that were subjected to early life stress (Waters et al., 2022), highlighting the need to consider sex in future studies of adult neurogenesis. Moreover, these and other data indicate that neurogenesis is often dispensible for behavior in naive animals but can contribute when animals are faced with additional stressors before, or during, testing (Snyder et al., 2011; Glover et al., 2017; Anacker et al., 2018).

It is typically understood that neurogenesis benefits cognition and so it may seem paradoxical that blocking neurogenesis improved water maze learning in females. However, it has been repeatedly demonstrated that males and females can display opposite patterns of hippocampal-dependent learning, with manipulations facilitating performance in males in some paradigms and facilitating performance in females in others (Luine, 2002; Conrad et al., 2004; Bangasser and Shors, 2007). Our findings also may seem paradoxical if it is assumed that “faster is better” in the water maze. It is increasingly well-documented that sex differences in learning tasks can reflect strategy differences rather than frank differences in learning ability (Shansky, 2018; Tronson, 2018), and we have previously found that focusing on escape latencies can mask neurogenesis-dependent differences in navigational choice preferences (Yu et al., 2019). Here, we found that male neurogenesis-deficient rats performed more general searches, but female neurogenesis-deficient rats tended to (nonsignificantly) perform more spatially-specific searches. While it is common to view spatially-specific searches as “better,” generalized search has clear advantages in cases where a spatial goal moves to a new or unexpected location (Steele and Morris, 1999; Richards et al., 2014). Thus, one possibility is that neurogenesis adjusts search/memory specificity differently, increasing it in males and perhaps decreasing it in females. That females trained at 16°C had higher levels of Fos in the dorsal DG indicates that there are clear sex differences in regional hippocampal recruitment, which could impact the adoption of precise search strategies that depend on this subregion of the hippocampus (Ruediger et al., 2012).

Another possibility, related to the fact that neurogenesis effects were selectively observed in 16°C water, is that emotional functions of neurogenesis were differentially engaged by stress. In other studies, stress impairs spatial learning in males and is either without effect, or actually improves learning, in females (Luine, 2002; Conrad et al., 2004). These divergent effects may reflect differential effects of stress on cognition (males) and hyperarousal (females; Bangasser et al., 2018). Since neurogenesis ablation mimics some features of the stressed brain (e.g., structural atrophy; Schloesser et al., 2013; Schoenfeld et al., 2017), male learning could have been impaired by dysregulated integration of stress and learning, and females may have learned faster because of heightened arousal and attention effects. A role for attentional processes is also suggested by recent work showing that blocking neurogenesis reduces orienting responses to distractor stimuli (Weeden et al., 2019), an effect that may explain why TK rats are faster to navigate a dry spatial maze in the presence of an aversive, but irrelevant, mint odor (Schoenfeld et al., 2021). Given sex differences in processing object arrays and configurations (Koss and Frick, 2017), blocking neurogenesis may differentially alter water maze cue processing such that females are less susceptible to distraction from irrelevant cues (leading to faster escape) but males are less attentive to relevant cues (leading to slower escape).

Finally, insights into the potential adaptive significance of neurogenesis also come from our analyses across temperatures (Fig. 7). Intact females were highly sensitive to temperature: 16°C shifted females away from random and wall-focused search, toward the center of the pool and the specific area of the platform. In contrast, TK females were not different at 16°C and 25°C. Thus, in females, neurogenesis promotes changes in strategy according to the aversiveness of the situation. In males, neurogenesis promoted equivalent strategy usage 16°C and 25°C, which could also be adaptive in cases where performance needs to remain stable despite perturbations from external forces.

### Sex differences in hippocampal subregional activation

To investigate possible subregional and cellular mechanisms we examined activity-dependent Fos expression along the dorsoventral axis in male and female rats that did, or did not, have adult neurogenesis. While previous studies have reported that ablating neurogenesis can increase (Burghardt et al., 2012; Drew et al., 2016; Anacker et al., 2018) or decrease (Glover et al., 2017; Seib et al., 2021) activity in the hippocampus, here we found no effect on global Fos expression among dentate granule cells. Newborn neurons also target inhibitory interneurons (Restivo et al., 2015; Drew et al., 2016), whose activity regulates the precision of hippocampal-dependent memory (Ruediger et al., 2011; Guo et al., 2018). However, we also observed no changes in inhibitory neuron recruitment in TK rats relative to WT rats. While these findings suggest that neurogenesis ablation did not affect behavior by altering hippocampal activity, it is possible that activity differences were present early in training, when sex and genotype differences were more prominent. It is also worth noting that these findings do not preclude changes to other forms of neuronal activity (for example, electrophysiological changes).

Little is known about how dorsoventral subregions of the hippocampus are activated in males and females by training in the standard spatial water maze. Here, we found that females consistently had greater levels of DG activity than males, particularly at 16°C. This was largely driven by elevated Fos levels in the dorsal hippocampus, a finding that builds on previous evidence that the spatial water maze recruits dorsal more than ventral DG (Snyder et al., 2009). However, whereas that study only included males, here we find that the dorsoventral gradient is significantly stronger in females. Notably, the opposite gradient was observed in GAD67^+^ inhibitory cells and in CA3 pyramidal cells. Since the temporal progression of water maze learning strategies involves sequential recruitment of ventral to dorsal hippocampus (Ruediger et al., 2012), we explored relationships between water maze performance (latency, path error, strategy specificity on acquisition and probe trials) and activity in the dorsal and ventral DG. However, we found no consistent correlations, suggesting that other forms of activity and plasticity may be more tightly linked to performance.

### Sex differences in morphologic plasticity of adult-born neurons

To our knowledge, this is the first study to examine functionally-relevant morphologic features of adult-born neurons in males and females. At baseline, we observed no differences in spine density or mossy fiber terminal size between the sexes. However, water maze training induced plasticity of excitatory synaptic structures but only in males. Since blocking neurogenesis impaired 16°C learning in males, 16°C-induced spinogenesis may be important for learning under stress in males, possibly allowing for greater association of sensory information from entorhinal cortical inputs. Somewhat surprisingly, training reduced the size of mossy fiber terminals in males. These findings are reminiscent of work showing that the CA3 pyramidal neuron apical dendrites, which are targeted by mossy fiber axons, undergo greater stress-induced plasticity in males than in females (Galea et al., 1997). Given the link between mossy fiber terminal size and synaptic strength (Galimberti et al., 2006, 2010), training may have reduced synaptic strength in male rats trained at 25°C, suggesting that new neurons in males may play a weaker role in memory under less aversive conditions. Likewise, we observed fewer filopodial protrusions in both males and females trained at 25°C, suggesting that new neurons are less likely to recruit inhibitory circuits in less aversive conditions, an effect that could reduce memory precision (Ruediger et al., 2011; Guo et al., 2018).

## Synthesis

Reviewing Editor: Karyn Frick, University of Wisconsin-Milwaukee Decisions are customarily a result of the Reviewing Editor and the peer reviewers coming together and discussing their recommendations until a consensus is reached. When revisions are invited, a fact-based synthesis statement explaining their decision and outlining what is needed to prepare a revision will be listed below. The following reviewer(s) agreed to reveal their identity: Natalie Tronson. Note: If this manuscript was transferred from JNeurosci and a decision was made to accept the manuscript without peer review, a brief statement to this effect will instead be what is listed below.

This manuscript is a revised version of a paper reviewed at the Journal of Neuroscience and transferred to eNeuro. The reviewers of the revised paper felt it provides interesting new data regarding potential sex differences in the role of new neurons in stress-modulated spatial learning and morphology of newborn cells in rats. Although it was felt that conclusions are limited by the model used, the data set was thought to be useful and appropriately interpreted. Overall, the reviewers thought that this is a much improved, much clearer version than the previous submission, and many of the major critiques were addressed. However, they felt that several issues still remain, as detailed below.

Before describing those issues, however, I would like to comment on the unusual way in which statistical analyses are presented and ask the Results be edited to provide a more conventional presentation of significant results. It appears that all statistical results are written into the Figure Legends and contained in the Extended Data file, leaving few in the Results section. As a reader, I find it frustrating and challenging to go searching for the statistical results on which the author’s conclusions rest, and prefer not to have to dig through figure legends of at least half a page. Typically, significant stats are located within the Results section as a way to support the author’s conclusions about the data. Thus, I request that you find a way to incorporate significant findings into the Results section if at all possible. If there is some compelling reason why this is not possible, then please make that case in your rebuttal. If you include the Extended Data file in your revision, then you must clearly indicate in the Results section which data are included in this file and make sure to reference the file throughout the Results. In addition, although I appreciate the transparency of the Extended Data file, it appears that at least some of its contents are already presented in graphical or text form in the figures and figure legends. If so, it is necessary to also include that information in the Extended Data file?

Additional reviewer comments are as follows:

1. “Critically, these disorders affect women to a greater extent than men, suggesting that 62 neurogenesis functions in stress may vary depending on sex and gender (Kessler et al., 2012)"

This is not logically consistent. Together with the data in rodents? Maybe?

2. One reviewer felt that Figure 1 should be deleted and the findings incorporated into the text of the manuscript. They thought that showing that information as a display item is more appropriate for a position paper than to provide rationale for a research article. On this point, I agree. If you do decide to publish this figure elsewhere, the other reviewer had the following questions/comments: “The sum of Male only, Female only, Male & Female, and Sex unspecified should add to 100%, correct? It would then make sense if “reported data by sex” was reported separately (or at least after the other measures) since this is a separate but related issue. In addition, when you say “reported data by sex” do you mean use sex as a variable, or do you mean disaggregated by noting male and females on individual data points/examining means/ranges for males and females separately? This would be an interesting statistics to add. The graph with the additional methods now makes sense. It is extremely frustrating to see that the proportion of studies using males only is roughly the same while the proportion studying “females only” decreases as studies including both sexes increasing. “

3. Figure 2 images do not reflect the quantitative data shown on the graphs. A very large difference in DCX cells is shown in the graphs and the images between groups look almost identical.

4. In Figure 3, noting on the figure which are cold and which are warm water tests would be helpful (or a key noting the meaning of the blue and red color coding)

5. Graphs showing sex differences would be more accurate if they were not shown with lines connecting the male and female data. This gives the impression of some continuity between the groups and is misleading. These data should be shown as bar graphs.

6. Individual data points should be shown for behavioral graphs.

7. The species should be mentioned in the title.

8. Throughout the paper, the authors refer to “neurogenesis” modulating effects. It seems overly general to say that a process is modulating an effect when what it seems they mean is that immature neurons are modulating the effect. The authors should consider changing this wording.

9. Line 578 - please temper the interpretation of fos-activity reflecting overall activity (here and elsewhere). You can say that decreased neurogenesis did not alter activation of fos, but not all neurons/activity increases fos. It’s suggestive, but not deterministic. Especially, as you note in your response to reviewer 1, that IEGs have different thresholds and likelihood for activation states.

## Author Response

Please see our responses below, in bold.

- Extended Data should be labeled as Figure 1-1, Figure 1-2, Table 1-1, etc., so they

indicate which figure they are supporting (i.e. Extended Data table supporting Figure 5

labeled as Figure 5-1).

- Extended Data figure/table should be referenced in the legend for the figure/table it is

supporting. Please add a reference to the Extended Data figure/table in the

corresponding main article figure/table.

In “Preparing a manuscript” [https://www.eneuro.org/content/preparingmanuscript] it states “Extended data that supports more than one figure and/or

table should be labeled as supporting the figure or table referred to first in the

text.” It sounds like this applies to our situation, since our extended data file

contains the data that supports all of the figures. We therefore refer to it as

Extended Data Fig. 1-1. We now state at the beginning of the results, and in the

legend for the first figure, that all of the underlying data and statistical analyses

for all figures can be found in this file. Is this an acceptable approach? This is

also how we provided, and referred to, the underlying data in Cole et al., 2020, J

Neurosci. Alternatively, we could break the data file up into discrete files that only

support individual figures but this would result in 11 different data files and

would make accessing the data more cumbersome.

Synthesis Statement for Author (Required):

This manuscript is a revised version of a paper reviewed at the Journal of Neuroscience

and transferred to eNeuro. The reviewers of the revised paper felt it provides interesting

new data regarding potential sex differences in the role of new neurons in stress-modulated spatial learning and morphology of newborn cells in rats. Although it was felt

that conclusions are limited by the model used, the data set was thought to be useful

and appropriately interpreted. Overall, the reviewers thought that this is a much

improved, much clearer version than the previous submission, and many of the major

critiques were addressed. However, they felt that several issues still remain, as detailed

below.

Before describing those issues, however, I would like to comment on the unusual way in

which statistical analyses are presented and ask the Results be edited to provide a

more conventional presentation of significant results. It appears that all statistical results

are written into the Figure Legends and contained in the Extended Data file, leaving few

in the Results section. As a reader, I find it frustrating and challenging to go searching

for the statistical results on which the author’s conclusions rest, and prefer not to have

to dig through figure legends of at least half a page. Typically, significant stats are

located within the Results section as a way to support the author’s conclusions about

the data. Thus, I request that you find a way to incorporate significant findings into the

Results section if at all possible. If there is some compelling reason why this is not

possible, then please make that case in your rebuttal.

We don’t have a perfect answer or solution for this since spreading results over

the text, figure and figure legend will always require one to navigate between

these 3 items (often on different pages) to fully absorb the data. We appreciate

that putting all of the statistics in the results text makes for a coherent package of

descriptive and statistical evidence. But visual inspection of the data itself is also

important for readers to assess conclusions that are present in the results text.

Typically, our preference (in papers we’ve published at SFN journals and

elsewhere) has been to keep the figures and analyses together, so one can

directly relate patterns that are visible in the data with their corresponding

statistical analyses. In some cases we have found that extensive statistical

reporting in the results text makes for challenging reading. And so this keeps the

results text easy to read. We don’t know which style is more common (and likely

there isn’t a one-size-fits-all approach here) but, in skimming our recently-read

articles, we often find statistical analyses in the figure legends (a couple of

examples are very recently published articles on sex differences that we have

now cited in the revised manuscript: Waters, Gould et al, 2022, Neurobiol Stress;

Le, Lynch et al, 2022, Nat Neurosci). And so, since making this change wouldn’t

solve the problem navigating between text, figure and legend, and since this style

preference doesn’t affect the validity of our analyses or interpretations (but it

would involve a fair bit of work), we would prefer to keep many of the statistical

analyses in the legend alongside the figures.

If you include the Extended Data file in your revision, then you must clearly indicate in

the Results section which data are included in this file and make sure to reference the

file throughout the Results. In addition, although I appreciate the transparency of the

Extended Data file, it appears that at least some of its contents are already presented in

graphical or text form in the figures and figure legends. If so, it is necessary to also

include that information in the Extended Data file?

We have put all of the underlying data in the Extended Data file and so in a sense

this duplicates what is present in the figures, except the actual data is more

precise. We feel it is important to include the actual data for reasons that are

aligned with eNeuro’s mission to promote statistical rigor and reproducibility. In

our original reviews we were asked to provide effect size measures. In

researching this practice we learned that there are many different types of effect

sizes, that some are better (eg less biased) than others, but that tools and

methods for calculating some effect sizes for certain experimental designs are

not yet readily available (eg see Lakens, 2013, Frontiers in Psychology). We

therefore opted to report partial eta squared because, while it is not perfect, it is

widely-used and easy to calculate. However, by providing the underlying data,

readers can compute their own effect size measurement as tools become

available and norms change. This may be particularly important for future metaanalytic studies of neurogenesis and sex differences, since effects are variable

across studies and theory is best assessed from a body of work.

In addition to the underlying data, the Extended Data file also contains the full

statistical analyses for all experiments. Some of these are duplicated in the

results text or figure legends. However, many are not duplicates and so for

consistency’s sake, and to provide a resource that allows readers to easily

access any of the statistical results (but minimally increases file size), we opted

to simply put all of the statistical results in the Extended Data file.

Additional reviewer comments are as follows:

1. “Critically, these disorders affect women to a greater extent than men, suggesting

that 62 neurogenesis functions in stress may vary depending on sex and gender

(Kessler et al., 2012)"

This is not logically consistent. Together with the data in rodents? Maybe?

Agreed, “together with the data from rodents” was what we originally intended

but this may have been lost as the rodent literature was discussed in the previous

paragraph.

We have now changed it to “Stress-related disorders such as anxiety, PTSD and

depression impact a substantial fraction of the population and these disorders

affect women to a greater extent than men. Together with the data from rodents,

this suggests that neurogenesis functions in stress may vary depending on sex

and gender (Kessler et al., 2012).”

2. One reviewer felt that Figure 1 should be deleted and the findings incorporated into

the text of the manuscript. They thought that showing that information as a display item

is more appropriate for a position paper than to provide rationale for a research article.

On this point, I agree. If you do decide to publish this figure elsewhere, the other

reviewer had the following questions/comments: “The sum of Male only, Female only,

Male & Female, and Sex unspecified should add to 100%, correct? It would then make

sense if “reported data by sex” was reported separately (or at least after the other

measures) since this is a separate but related issue. In addition, when you say “reported

data by sex” do you mean use sex as a variable, or do you mean disaggregated by

noting male and females on individual data points/examining means/ranges for males

and females separately? This would be an interesting statistics to add. The graph with

the additional methods now makes sense. It is extremely frustrating to see that the

proportion of studies using males only is roughly the same while the proportion studying

"females only” decreases as studies including both sexes increasing. “

We removed this figure from the manuscript and have published it on Figshare

(https://figshare.com/articles/figure/Adult_neurogenesis_studies_primarily_use_

males/19319849) and now only refer to it in the text. For the record, we liked the

idea of putting the “reported data by sex” after the other measures.

3. Figure 2 images do not reflect the quantitative data shown on the graphs. A very

large difference in DCX cells is shown in the graphs and the images between groups

look almost identical.

Good catch. In making the high resolution figures somehow the WT image was

duplicated in place of the TK image. This has been fixed.

4. In Figure 3, noting on the figure which are cold and which are warm water tests would

be helpful (or a key noting the meaning of the blue and red color coding)

This has been added to the figure (large text on top of each group of graphs that

states “16C” or “25C”).

5. Graphs showing sex differences would be more accurate if they were not shown with

lines connecting the male and female data. This gives the impression of some continuity

between the groups and is misleading. These data should be shown as bar graphs.

This has been changed.

6. Individual data points should be shown for behavioral graphs.

This has been changed (except for data over the 3 days of training, since the

graphs become too muddled).

7. The species should be mentioned in the title.

Done.

8. Throughout the paper, the authors refer to “neurogenesis” modulating effects. It

seems overly general to say that a process is modulating an effect when what it seems

they mean is that immature neurons are modulating the effect. The authors should

consider changing this wording.

Done.

9. Line 578 - please temper the interpretation of fos-activity reflecting overall activity

(here and elsewhere). You can say that decreased neurogenesis did not alter activation

of fos, but not all neurons/activity increases fos. It’s suggestive, but not deterministic.

Especially, as you note in your response to reviewer 1, that IEGs have different

thresholds and likelihood for activation states.

Agreed, and we have added another statement to this effect, on the fact that there are other

forms of neuronal activity that could have changed/could be investigated.
